# hadge: a comprehensive pipeline for donor deconvolution in single-cell studies

**DOI:** 10.1186/s13059-024-03249-z

**Published:** 2024-04-26

**Authors:** Fabiola Curion, Xichen Wu, Lukas Heumos, Mylene Mariana Gonzales André, Lennard Halle, Matiss Ozols, Melissa Grant-Peters, Charlotte Rich-Griffin, Hing-Yuen Yeung, Calliope A. Dendrou, Herbert B. Schiller, Fabian J. Theis

**Affiliations:** 1 Institute of Computational Biology, Computational Health Center, Helmholtz Munich, Neuherberg, Germany; 2 Department of Mathematics, School of Computation, Information and Technology, Technical University of Munich, Garching, Germany; 3 Nuffield Department of Medicine, Wellcome Centre for Human Genetics, University of Oxford, Oxford, UK; 4 Comprehensive Pneumology Center, German Center for Lung Research (DZL), Munich, Germany; 5 Nuffield Department of Orthopaedics, Rheumatology and Musculoskeletal Sciences, The Kennedy Institute of Rheumatology, University of Oxford, Oxford, UK; 6 TUM School of Life Sciences Weihenstephan, Technical University of Munich, Freising, Germany; 7 Wellcome Sanger Institute, Hinxton, UK; 8 School of Cell Matrix and Regenerative Medicine, The University of Manchester, Manchester, UK; 9 Research Unit Precision Regenerative Medicine, Helmholtz Munich, Neuherberg, Germany; 10 Institute of Experimental Pneumology, LMU University Hospital, Ludwig-Maximilians University, Munich, Germany

**Keywords:** Single-cell, Donor deconvolution, Genetic, Hashing, Nextflow

## Abstract

Single-cell multiplexing techniques (cell hashing and genetic multiplexing) combine multiple samples, optimizing sample processing and reducing costs. Cell hashing conjugates antibody-tags or chemical-oligonucleotides to cell membranes, while genetic multiplexing allows to mix genetically diverse samples and relies on aggregation of RNA reads at known genomic coordinates. We develop hadge (hashing deconvolution combined with genotype information), a Nextflow pipeline that combines 12 methods to perform both hashing- and genotype-based deconvolution. We propose a joint deconvolution strategy combining best-performing methods and demonstrate how this approach leads to the recovery of previously discarded cells in a nuclei hashing of fresh-frozen brain tissue.

**Supplementary Information:**

The online version contains supplementary material available at 10.1186/s13059-024-03249-z.

## Background

Single-cell RNA sequencing (scRNA–seq) technologies have unlocked unprecedented resolution to discover complex mechanisms of health and disease in human biology [1]. Droplet-based methods, which encapsulate aqueous cells into oil constituting a micro-chamber for lysis and retrotranscription of the RNA of individual cells, have made single-cell sequencing more accessible and dramatically increased the throughput of single cells from individual samples [2]. The cDNA produced in these reactions is uniquely barcoded for each droplet, such that the retrieval of these barcodes enables the association of sequencing readouts to individual cells. Despite considerable strides made in cellular profiling methods, the application of scRNA-seq to biomedical studies and clinical applications, which often require complex multi-sample and multi-condition experiments, has been limited by sample throughput, cost, and susceptibility to technical variability [2]. When samples cannot be acquired fresh or immediately processed after the acquisition, as may be the case for biobank specimens, fixation techniques that allow to preserve the biological material and optimize single-cell and nuclei profiling via multiplexing are a viable option [3–6]. In recent years, methods have emerged that allow the pooling of single cells and nuclei from individual samples [7], often relying on multiplexing techniques [8]. These methods have found wide applicability [8] and are now routinely used to carry out population-scale studies with single-cell sequencing protocols [9, 10].

**Fig. 1:**
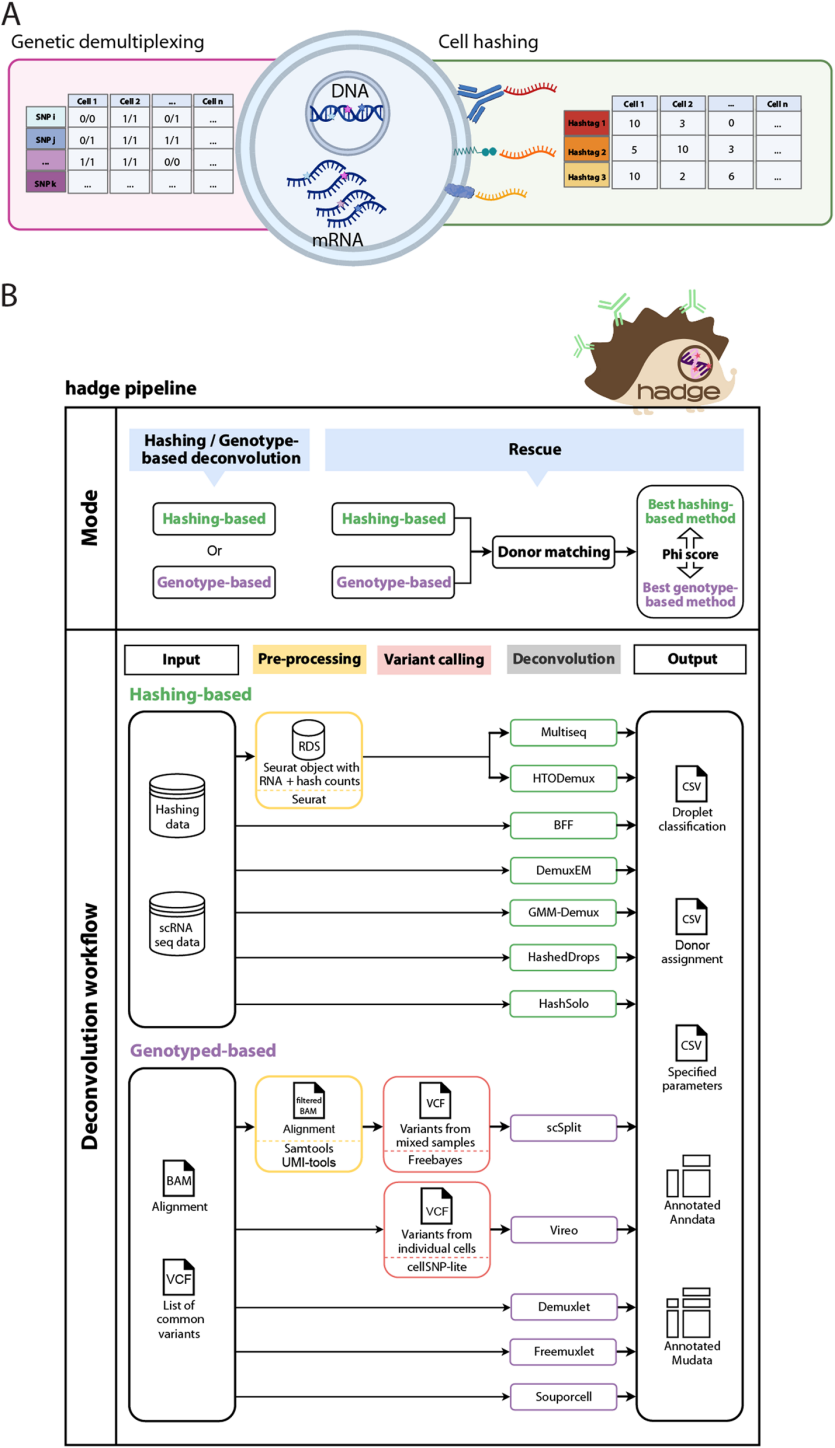
Overview of donor deconvolution and the hadge pipeline. **A** Schematic example of the cellular components leveraged by single-cell multiplexing experiments. Hashing cell counts and scRNAseq reads with SNP calling by cell are the input to the hadge deconvolution pipeline. **B** hadge implements 12 methods across two sub-workflows of which seven are hashing-based and five are genotype-based deconvolution that can be run independently, in parallel or jointly, in rescue mode. In rescue mode, the pipeline offers the option to refine hashing results with genotype-based deconvolution methods to rescue failed hashing experiments in the donor-matching process. It compares the concordance in donor identification between hashing and genotype-based methods and identifies the best pair of two strategies based on the calculated Phi score

To date, there are two major protocols to generate a mixture of cells from multi-sample studies: “cell hashing” and “genotype-based multiplexing.” Cell hashing is a sample processing technique that tags the membrane or nuclei of cells in individual cell-suspension samples with unique oligonucleotide barcodes. One option is to stain the individual samples with oligonucleotide-labeled antibodies that target proteins ubiquitously expressed on the cell or nucleus surface [11]. Another option is to chemically conjugate oligonucleotides directly to the membrane constituents, for example by hybridization of a lipid-modified oligonucleotide (LMO) to the hydrophobic cell membrane, a technique called “lipid tagging” [12], or by chemical ligation of the oligonucleotide to exposed N-Hydroxysuccinimide-reactive amines, a technique called “chemical barcoding” [11, 13, 14]. After staining or tagging, cells undergo a washing or quenching process, allowing for the safe combination of different samples into a single mixture in one tube. From this mixture, two separate sequencing libraries are created: one for single-cell RNA (scRNA) and one for hashing oligos (HTO). These libraries are independently sequenced to yield two distinct single-cell count matrices, corresponding respectively to scRNA and HTO data. To deconvolve the cell’s source sample, the HTO counts are processed to discover cell barcodes positive for at least one hashtag, using cell-hashing deconvolution methods [11, 12, 15, 16]. Cell barcodes are classified into “Singlets,” if they are positive for one tag; “Doublets,” if positive for two or more; and “Negatives,” when only a low background-noise signal is detectable (Fig. 1A). Cell-tagging approaches suffer from constraints such as low starting cell numbers, as these methods require washing steps that may result in cell-number loss. Furthermore, different issues can impair the quality of a hashing experiment and therefore decrease the final number of uniquely identified cells. Antibodies or free oligonucleotides can persist in suspensions if an adequate number of washes is not performed, or can attach to debris from membrane lysis in fixed samples [15].

Genotype-based multiplexing allows the mixing of samples with unique genetic composition, where natural genetic variants serve as inherent cell barcodes [17]. Users can harness these genetic barcodes to determine the identity of each cell in the mixture. Provided a genotype reference, the scRNA reads are scanned for single nucleotide polymorphisms (SNPs) in the reference, and a table of SNP assignment to cells is produced to computationally infer the donors (Fig. 1A). Cell barcodes with a genetic composition matching one donor are called “Singlets,” cell-barcodes with a mixture of at least two donors genotypes are deemed “Doublets,” and cells where the read coverage is insufficient to identify their genetic composition are “Negatives.” One limitation of this approach is the need to rely on additional data to correctly assign the cell mixtures. Users can genotype the individual samples through SNP arrays or bulk RNA-seq followed by variant calling and then aggregate the expression values at these genomic positions for deconvolution. The same process can be conducted without genotype of origin or “genotype-free,” by piling up the mixture of scRNA onto an unrelated genomic reference of genotypes such as that provided by the “1000 Genome Project.” However, this approach can only deconvolve the cell mixture in the form of anonymous donors and additional processing is needed to match them to the sample of origin.

The limitations of each of these protocols can be mitigated when combining demultiplexing approaches. Experiments, where the hashing libraries are of low quality, can be rescued and successfully demultiplexed using the natural genetic variation of their RNA libraries. The combination of hashing and genetic deconvolution methods represents a viable option for combinatorial experimental design and can result in increased cell recovery rate and calling accuracy [18]. Moreover, joint demultiplexing can be a cost-effective deconvolution strategy as it further avoids having to produce sample-specific genotyping data in the form of SNP arrays or bulk sequencing methods for variant calling [18]. To date, at least nine hashing and five genotype-based deconvolution methods have been developed, each with unique strengths and weaknesses [12, 15, 17, 19–22]. However, investigations on joint demultiplexing strategies have been limited to the combination of two specific tools instead of computationally testing the best combination of demultiplexing methods, therefore neglecting the utility of other widely used tools [18]. Although single workflows for hashing-based deconvolution and genotype-based deconvolution exist [23, 24], no study has combined all the tools for both approaches in a single comprehensive and efficient pipeline, such that both hashing and genotype deconvolution pipelines can be run in parallel on multiple samples, providing a score to discover the best methods across the board, with the final goal of maximizing the number of rescued cells and increasing the confidence of deconvolution.

Therefore, there is a critical need for a unified pipeline that integrates the strengths of multiple donor deconvolution tools. Here we present the hadge (hashing deconvolution combined with genetic information) pipeline. Our Nextflow-based pipeline [25] enables deconvolving samples of both hashing and genetic multiplexing experiments either independently or simultaneously. hadge allows for the automatic determination of the best combination of hashing and SNP-based donor deconvolution tools. Moreover, hadge provides a rescue mode to run both genetic and hashing approaches jointly to rescue problematic hashing experiments in cases where donors are genetically distinct. We demonstrate our pipeline using a single nuclei hashing experiment of fresh frozen multiple sclerosis (MS) brain tissue and show that joint deconvolution allows us to rescue high-quality cells that would have been otherwise discarded. Finally, we benchmark our pipeline with the state-of-the-art tools and a large-scale scRNA dataset [9].

## Results

### The hadge pipeline

Hadge offers a user-friendly, zero-config solution for analyzing multiplexed single-cell data at scale (Fig. 1B). Our pipeline takes advantage of Nextflow’s cloud-computing capabilities, enabling efficient use of cloud resources to accelerate the analysis of large datasets. Furthermore, Nextflow’s built-in containerization functionality simplifies deployment, providing a more reliable and reproducible analysis environment. The hadge pipeline consists of 12 deconvolution tools, including five genetics-based tools (Demuxlet [17], Freemuxlet [26], Vireo [22], scSplit [20], and Souporcell [21]), seven hashing-based tools (HTODemux [27], Multiseq [12], HashedDrops [28], Demuxem [15], gmm-demux [29], BFF [24], and Hashsolo [30]), and one doublet-detection method (Solo [30]). All of these tools have been benchmarked in independent publications and are widely used by the scientific community [14, 23, 31, 32]. Furthermore, for methods that require additional preprocessing such as normalization of the HTO counts or variant calling, the hadge pipeline includes a preprocessing step before the genotype-based deconvolution algorithm is applied.

The hadge pipeline has three modes: “genetic,” “hashing,” and “rescue.” In the genetic or hashing mode, the pipeline runs either the genotype- or hashing-based deconvolution workflow allowing for choice of methods and customization of input parameters. Each of these pipelines can be run in parallel across multiple samples, reducing the time and effort required for deconvolution. Finally, in the rescue mode, hadge allows jointly deconvolving hashing experiments with genotype-based deconvolution tools, with the option to recover cells from failed hashing. Lacking prior individual genetic profiles that associate SNPs to explicit donors, genotype-based deconvolution tools assign cells to anonymous donors. Hadge de-anonymizes the donors by calculating the best match between a hashing and a genetic demultiplexing method. After the conversion of the cell deconvolution assignments into a binary matrix with rows representing cell barcodes and columns representing the assigned donors or hashtags, donor genotypes are matched with hashtags by measuring the concordance of two methods in assigning the droplets, computing pairwise Pearson correlation to determine the optimal match, hereby termed “Phi score” (see the “ Methods” section). hadge then generates a new assignment of the cells based on this optimal match between hashing and genotype-based deconvolution to uncover the true donor identity of the cells effectively rescuing cells from failed hashing with a valid genotyped-based deconvolution assignment. Finally, hadge outputs the results of the donor deconvolution for all combinations of methods and hyperparameters tested, both as a separate tabular format and as cell metadata in either Anndata [33] or MuData [34] objects, depending on the users’ choice.

### Hashing-based methods’ performance greatly varies with noisy HTO libraries

**Fig. 2:**
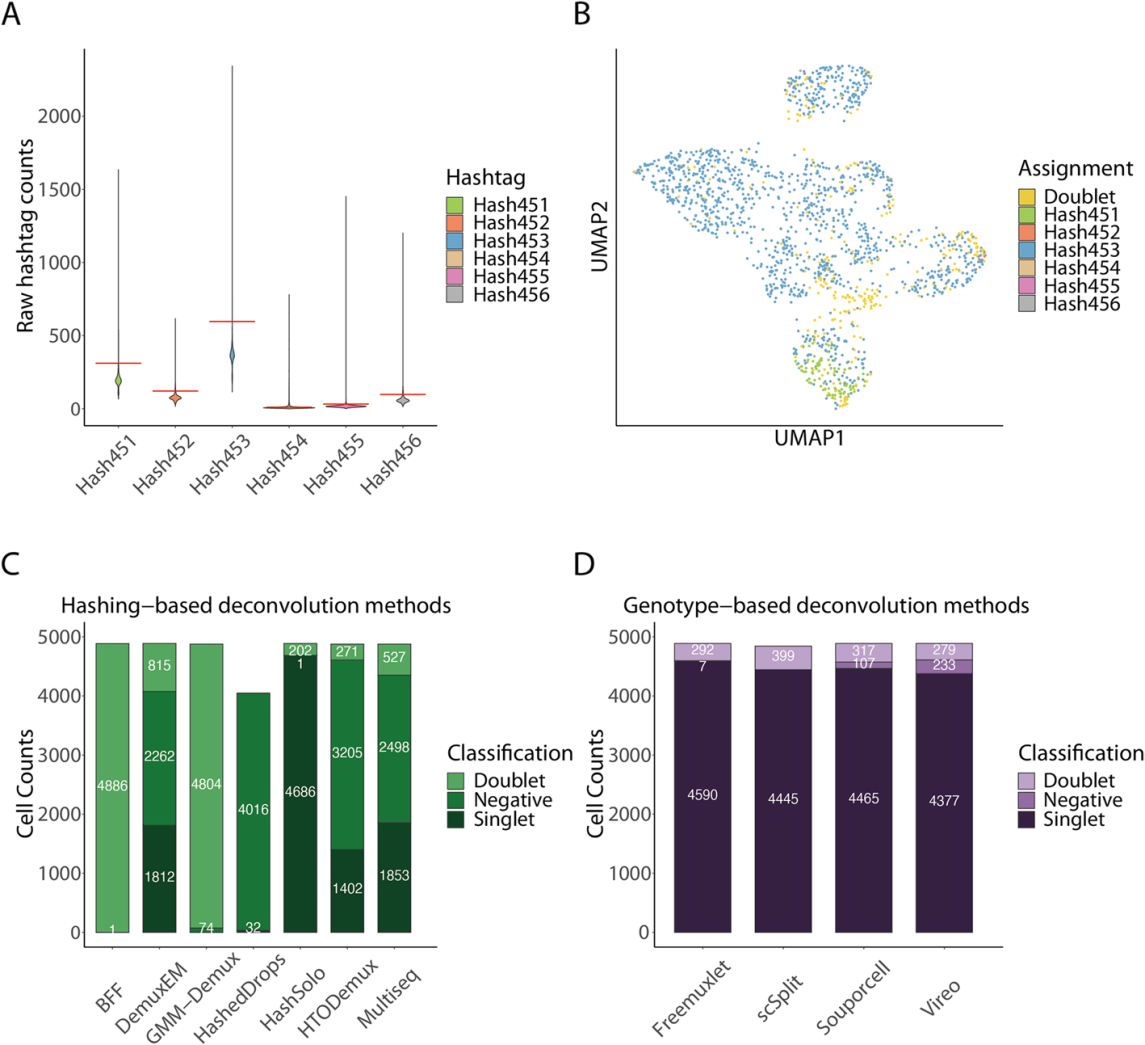
Comparison of the performance of donor deconvolution methods. **A** The violin plot of raw HTO counts shows high count levels of Hashtag 453 in cells with noisy or undetectable expression of the other HTOs. **B** UMAP visualization of normalized HTO counts colored by HTODemux assignment shows poor separation of the cells based on hashtags, with most droplets assigned to Hashtag 453. **C** Hashing-based deconvolution methods show the inconsistent assignment of cells, reported as the different proportions of cells identified as one of either singlet, negative, or doublet. **D** Genetic deconvolution tools show a more consistent assignment of the cell mixture to singlets, doublets, and negatives

**Fig. 3:**
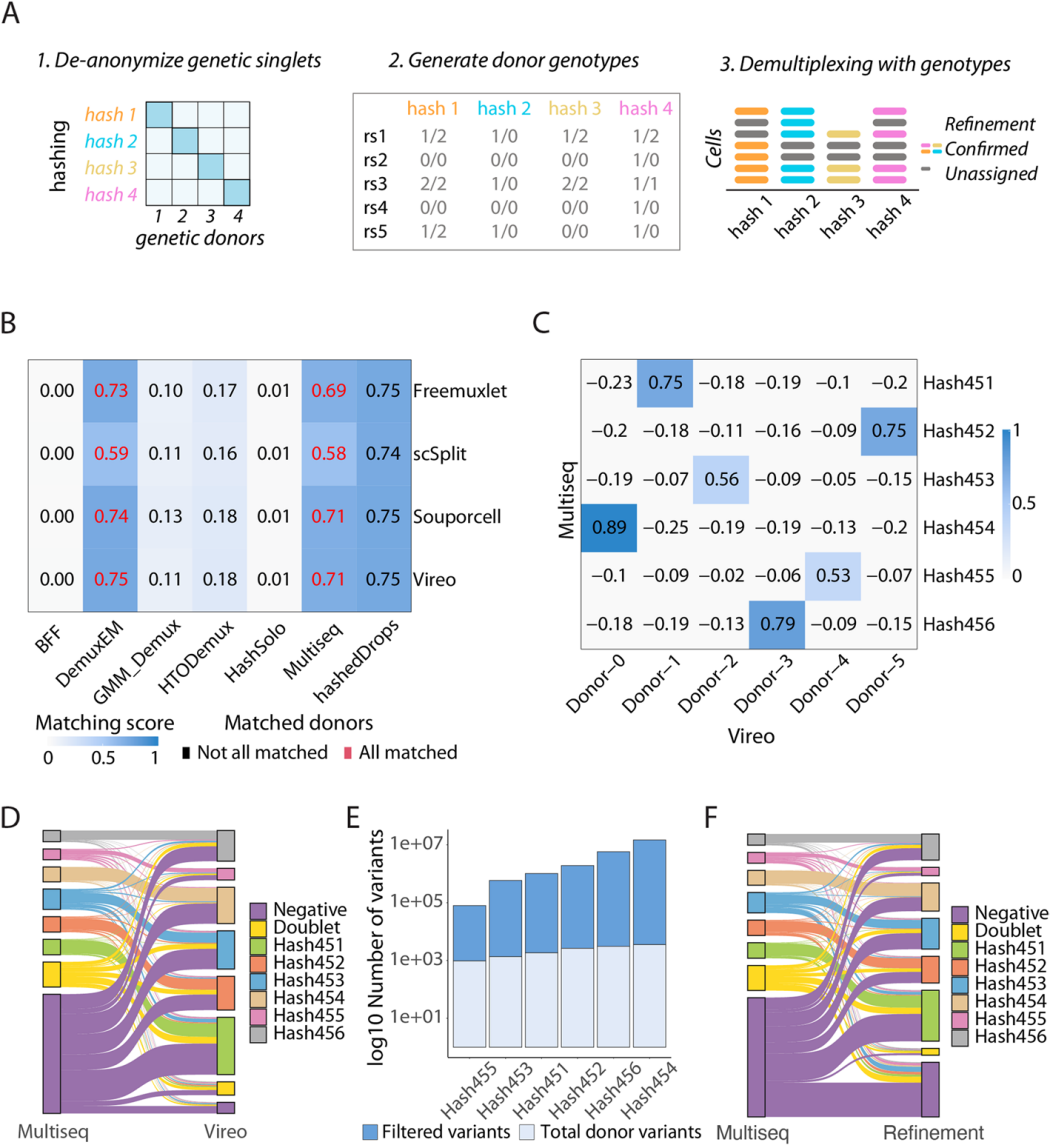
Joint deconvolution recovers high-quality cells. **A** Overview of the steps to extract cell variants from common SNPs in the population based on the assignment of Multiseq and Vireo. **B** Heatmap summarizing the donor matching result shows that DemuxEM and Multiseq are in high concordance with all genotype-based deconvolution methods, where all the donors are matched with a high matching score. **C** Correlation heatmap of donor identification between Vireo and Multiseq. **D** Sankey plot summarizing the percentages of cells deconvoluted by hashing (Multiseq) and after the joint deconvolution step (Vireo). **E** Number of donor-specific variants used as input for the refinement step. **F** Sankey plot summarizing the percentages of cells deconvoluted by hashing (Multiseq) and after the refinement step

We applied the hadge pipeline to a hashing dataset of single nuclei sequencing collected from post-mortem brain tissue from multiple sclerosis donors [35]. The hashing count matrix of this dataset presented a high background noise from non-specific antibody binding, which originally resulted in a high number of doublets and negative cells (Fig. 2A, B). We ran both hashing and genetic deconvolution workflows to assess the performance of the two types of approaches. We observed inconsistent hashtag counts (Fig. 2A, B and Additional file 1: Fig. S1). Specifically, hashtag 453 showed a high overall expression, while hashtags 454 and 455 were expressed in relatively low levels (Fig. 2A and Additional file 1: Fig. S1, S6). Due to the variable readout of the hashing oligos, the sample assignment of the hashing-based methods was not consistent. The number of detected singlets varied greatly between different methods (Fig. 2C and Additional file 1: Fig. S1-2, S7). While Hashsolo classified almost every droplet as a singlet, HashedDrops detected only 32 singlets among 4048 non-empty droplets. Notably, DemuxEM and Multiseq exhibited nearly identical performance (Additional file 1: Fig. S1-2,S4), both assigning nearly 1800 singlets, (Additional file 2: Table S1) with Multiseq identifying slightly more singlets and being considerably faster than DemuxEM. (Additional file 3: Table S1). Despite the noisy HTO readouts, the RNA profiles of these cells are still of good quality, allowing demultiplexing to be performed from the RNA library. Since the donor-specific reference genotypes are not available for this experiment, we run all genetic deconvolution tools in reference genotype-free mode. Compared to hashing, genotype-based deconvolution methods performed more consistently and identified significantly more singlets (Fig. 2D and Additional file 1: Fig. S3, S8-9). Each tool classified over 90% of the droplets as singlets, and there was consistent agreement between all tools for 3914 singlets (Fig. 3D). However, scSplit identified 296 droplets as doublets, which were consistently identified as singlets by three other methods. Due to the high consistency among Vireo, Freemuxlet, and Souporcell, and available benchmarks showcasing its favorable performance compared to the other tools [23], we decided to use Vireo as a baseline for genotype-based deconvolution methods.

### Joint deconvolution recovers cells with low-quality hashing data

Beyond determining the optimal combination of hashing- and genotype-based deconvolution methods, hadge aims to rescue cells whose hashing quality was low or whose hashes were missing (Fig. 3A). Hadge performs joint deconvolution with both hashing and genetic deconvolution tools to rescue high-confidence singlets. Only cells that can be confidently genetically deconvoluted are eligible to be rescued. After having demultiplexed the experiment in genotype-free mode [20, 22], the anonymous donors need to be matched to their original sample to be identified. Here, we rely on the hashing deconvolution to provide the known correspondence between the antibody hashtags and the original sample. Cells that are jointly deconvolved provide the key to de-anonymize the genetically rescued cells.

We first define the hashing method that matches the genetic demultiplexing method by calculating the Phi score (see the “ Methods” section). For each pair of hashing and genetic deconvolution outputs, we compute the pairwise Pearson correlation on the binarized cell classification vectors, thereby matching donors where a high correlation is observed. We then compute the matching score by summing the non-negative correlations and dividing by the number of expected donors, obtaining the degree of consistency in donor identification between any two methods. Based on the observed high matching score and the successful matching of all anonymous donors (Additional file 1: Fig. S4-5, S10), two hashing demultiplexing methods performed best compared to Vireo, namely Multiseq and Demuxem, both recovering identical matches between genetic and hashing donors (Fig. 3B, Additional file 1: Fig S4-5 and Additional file 2: Table S1). When the optimal match is identified, the identities recovered using the cells in the intersection between genetic and hashing can be propagated to the cells that are identified by genetic deconvolution alone. Here, we decided to use the joint demultiplexing of Multiseq and Vireo to showcase the rescue mode because of Multiseq’s reduced runtime. For every anonymous donor recovered by Vireo, there was only one hashtag with a high correlation, with scores ranging from 0.53 to 0.89 (Fig. 3C). Using the cells that are jointly deconvoluted into singlets by hashing and genetic demultiplexing, we extended the classification to those cells whose hashing was undetectable (negatives). We identify 90% of the cells as singlets, rescuing 89.7% of the original negatives (Fig. 3D), and double the number of recovered singlets for the hashes with the lowest detection rate (Hash454-456, Fig. 2A). Vireo is implemented to rely on cellSNP, which outputs the recovered genetic variants in each cell. We implemented an optional process in hadge to refine and confirm the quality of the deconvolution, by extracting cell-variants to reconstruct minimal donor genotypes from the common SNPs in the populations. Variants with low coverage (allele depth < 10) or a low frequency of the overrepresented allele (frequency < 0.1) were excluded, revealing 7866 variants that were unique to each donor (Fig. 3E). Since only a small fraction of the hashing-recovered singlets is sufficient to de-anonymize the genetic-singlets, we can use these reconstructed genotypes to run an additional genetic demultiplexing. Therefore, this final refinement step allows to effectively demultiplex cell mixtures without having to generate new SNP references. Using this refining approach, we identify 75% of the cells as singlets, with the number of rescued negatives decreasing to 69.7% (Fig. 3F). Nevertheless, we obtain 97.6% consistent donor assignment between the rescued and the refined assignments (Additional file 1: Table S1), suggesting that these variants were crucial in distinguishing a donor cluster from others during deconvolution.

### Recovered cells recapitulate known cell types

**Fig. 4:**
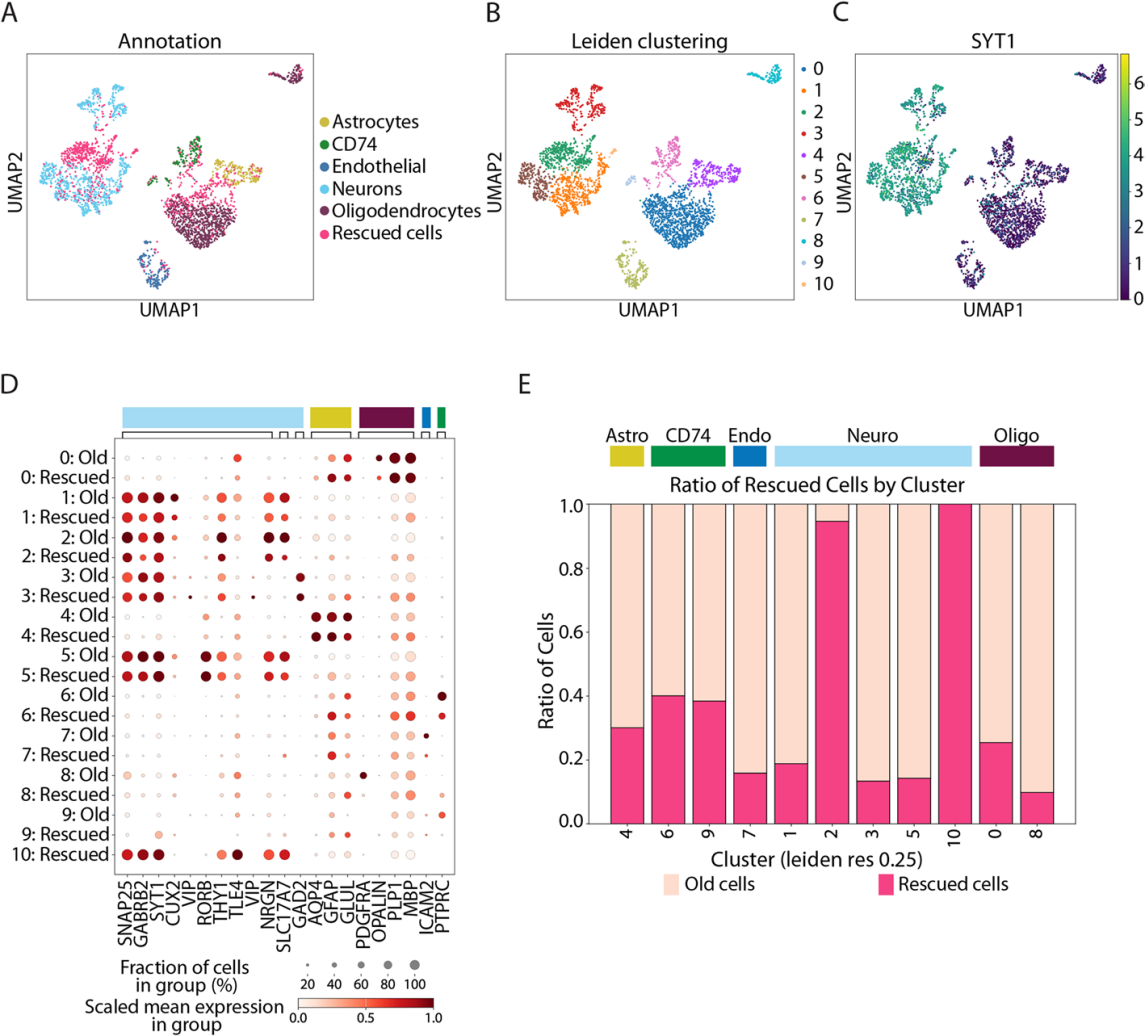
Recovered cells recapitulate known cell types. **A** UMAP of the single-cell gene expression data with old and rescued cells. **B** Leiden clustering of the dataset with old and rescued cells. **C** SYT1 expression defines rescued cells as a new cluster of neurons. **D** Dotplot of a selection of marker genes shows concordant expression of markers in old and rescued cells. **E** Barplot showing the cluster composition in old and rescued cells, with two neuronal clusters enriched for rescued cells. Colors on top of the barplot identify the cell annotation from **A**

To investigate whether the cells that are rescued are of good quality and biologically relevant, we reanalyzed the MS samples, including the recovered cells. We first merged the already existing annotation of the cells with the deconvolution information obtained from the hadge pipeline. We then applied quality filtering, removing cells based on gene content, mitochondrial percentage, and doublet rates (Additional file 1: Fig S12) (see the “Methods” section), reproducing the quality control performed in the original study but with a more stringent doublet detection threshold. With this approach, we retained 3208 cells, rescuing 952 cells that were excluded in the original study. We then embedded the cells using UMAP and calculated Leiden clustering. Most of the rescued cells were distributed across existing clusters, with comparable marker expression between the old and new cells (Fig. 4A, B, D, Additional file 1: Fig. S11). Intriguingly though, the percentage of rescued cells per cluster varied. While most of the clusters consisted predominantly of previously annotated cells mixing with a smaller part of rescued cells, two clusters were composed of more than half or even 100% rescued cells (Fig. 4E). While the smaller one of these, consisting solely of rescued cells, had an almost exclusively high expression of the marker *HTR2C*, we found the gene marker expression of, e.g., *SYT1*, *SLC17A7*, and low *GAD2* to be consistent with a neuronal profile with excitatory and non-inhibitory properties in both clusters. Reassuringly, the latter marker expression was in accordance with that of known neuronal clusters [36, 37] (Fig. 4C, D, Additional file 1: Fig. S11).

### Benchmarking hadge’s runtime and robustness

**Fig. 5:**
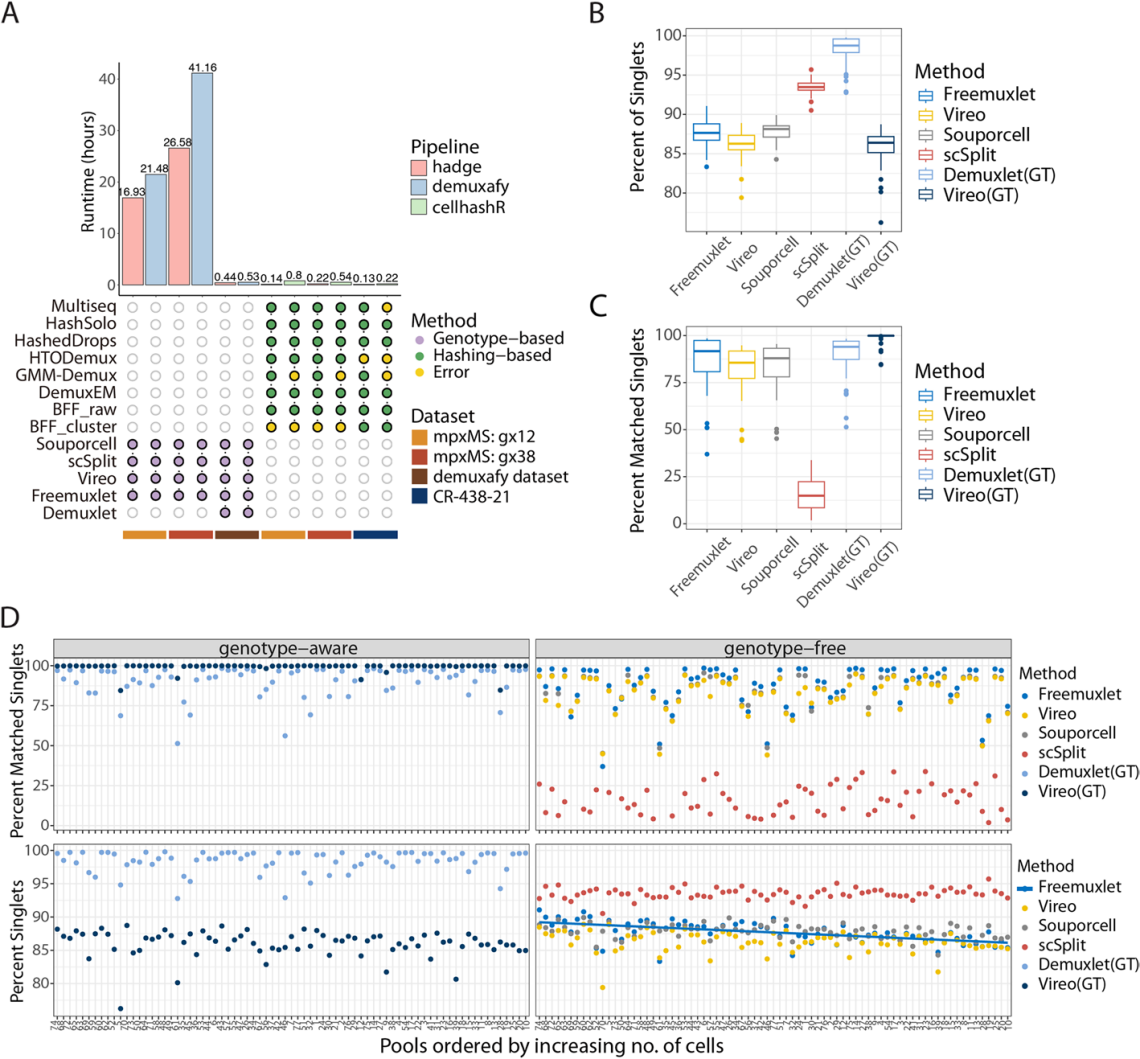
Benchmarking performance. **A** Hadge genetic and hashing demultiplexing pipelines were benchmarked against demuxafy and cellhashR. The benchmark was performed on three samples for each pairwise comparison, for a total of four samples (mpxMS:gx12, mpxMS:gx38, demuxafy dataset, CR-438–21 dataset). **B** Results of hadge genetic on the onek1k cohort; each boxplot represents the distribution of percentage singlets identified across 75 pools by each genetic deconvolution tool. **C** Percentage of correctly matched singlets for each tool; each boxplot represents the distribution across 75 pools. **D** Dotplot showing the effect of the number of cells per pool on the percentage of recovered and matched singlets. The regression line represents the fit of a linear model on the percentage of singlets identified by Freemuxlet (*R*.^2^ 0.35, p.adj < 0.0001)

To demonstrate the robustness and superior runtime of our proposed pipeline, we benchmarked its performance against two existing pipelines, demuxafy and cellHashR (Table 1). We submitted each pipeline on a Linux server with 32 CPU cores and 160 GB of RAM memory. In all benchmarks, hadge showed superior performance with respect to the optimization of computational resources and runtime (Fig. 5A). Both hadge-genetic and *demuxafy* successfully executed all methods for the two mpxMS samples and an additional dataset. However, in the hashing deconvolution of the mpxMS data, some methods (*bff_cluster, bff_raw*) ran but failed to deconvolve the cells in both hadge-hashing and *cellhashR*. Additionally, one method (*demuxmix*) failed to initialize in both pipelines and as a standalone method. Hence, we excluded *demuxmix* from hadge. Notably, despite successfully running *gmm_demux* within *hadge-hashing* or when called outside the pipeline, we were not able to run cellhashR’s *gmm-demux* module.

**Table 1:** Comparison of donor deconvolution pipelines

	Demuxafy	cellHashR	HTOreader	hadge
Framework	Singularity	R	R	Nextflow
Available genotype-based methods	5	-	Souporcell	5
Available hashing-based methods	-	7	HTOreader	7
Doublet detection methods	7	-	-	1
Concatenating	-	-	-(*)	+
Parallelized	-	-	-	+
Pre-processing tools	Samtools (*)	ProcessCountMatrix, PlotNormalizationQC	HTOClassification	Samtools
Variant calling tools	Freebayes(*), cellSNP-lite (*)	(not relevant for hashing-based)	(not relevant for hashing-based)	Freebayes, cellSNP-lite
Associating clusters and donors	Only through reference SNP genotypes	(not relevant for hashing-based)	From hashtags to donors based on confusion matrix	From hashtags to donors based on matching Phi score
Combining results	+	+	-	+
scverse compatibility	-	-	-	+

The “Pre-processing tools” and “variant calling tools” columns specify the respectively used tools that are (optionally) run before the deconvolution tools. “Concatenating” refers to the functionality to concatenate hashing-based and genotype-based deconvolution methods. “Combining Results” refers to the functionality that allows the merging of results from multiple methods into a single data frame during a single run, based on the users’ choice. ( +) The pipeline supports the mentioned functionality. ( −) The pipeline does not support the mentioned functionality. (*) The software is required as part of additional preprocessing outside of the pipeline

Next, we leveraged hadge’s fast multi-sample, multi-process handling to investigate how the input number of cells affects the performance of genetic and hashing demultiplexing. We ran hadge with default parameters on the onek1k dataset, which comprises 75 pools of cell mixtures from 9 to 15 donors each [9] and has ground truth donor-genotypes available. All tools detected a proportion of singlets per pool between 75 and 98% (Fig. 5B). However, when looking at matched donors within the singlets, the performance of the tools varied substantially, with scSplit having the lowest concordance with the original donor identities, while Demuxlet had the best performance in terms of recovered singlets and Vireo recovering the most matched singlets (Fig. 5C). We investigated if these two metrics were associated with the number of cells in each pool. Only Freemuxlet’s singlets percentage had a significant association with the number of cells per pool (*R*^2^ = 0.35, p.adj < 0.0001) (Fig. 5D), but all methods were significantly affected by the number of donors in the pools (Additional file 1: Fig. S13A, Additional file 3: Table S2), with the highest concordant calls reached on pools with 14 multiplexed donors (Additional file 1: Fig. S13B). Across all the pools, Vireo, Demuxlet, Freemuxlet, and Souporcell were mostly consistent, confirming these tools’ superior performance, and as observed on the MS dataset (Figs. 2 and 3). Additionally, we ran downsampling on two hashing and one genetic multiplexing experiments and used hadge to obtain the percentages of correctly assigned singlets. The hashing methods showed an overall similar trend with the percentage of matched singlets decreasing with the number of cells, except for HTODemux and Demuxem (Additional file 1: Fig. S14). All the hashing demultiplexing tools were able to correctly assign at least 90% of the cells after downsampling. The performance of the genetic demultiplexing tools was in line with what we observed on the onek1k dataset, and consistent for each tool across the different subsamples, with Vireo having the best performance across the board (~ 99% recovered matching singlets) (Additional file 1: Fig. S14). Hadge allowed us to efficiently benchmark the demultiplexing performances of all the methods across the two workflows. Collectively, these results indicate that the number of donors and cells in the cell mixtures can significantly affect the number of recovered cells and the quality of the deconvolution for both families of demultiplexing methods.

## Discussion

Single-cell multiplexing techniques enhance sample throughput, reduce costs, minimize technical variation, and improve cell type identification in single-cell genomics studies by increasing the number of samples and therefore reducing the gene expression variation associated with single-cell RNA sequencing. Some of the techniques for generating multiplexed single-cell mixtures require additional processing steps, which can introduce technical noise and result in a low yield of usable data. Furthermore, computational donor deconvolution errors can occur due to technical noise or experimental artifacts, leading to the misidentification of cells or barcodes.

We developed hadge, a comprehensive pipeline for donor deconvolution experiments generated with both genetic and hashing multiplexing methods. hadge is the only pipeline capable of processing both types of data inputs allowing for fine tuning of deconvolution experiments and performs favorably compared to the state-of-the-art pipelines. We leveraged the optimized multi-sample handling implemented in hadge to investigate the demultiplexing performance of the 12 demultiplexing methods included with varying numbers of cells and donors in the input cell mixtures. We showed that the different numbers of input cells and donors can significantly affect the performance of the tools, and users may need to take this into account when designing their experiment and interpreting the deconvolution results. As different tools rely on varying hyperparameters, it is possible to tune them to investigate the effect on cell deconvolution. To ensure confident identification of cell mixtures, hadge enables complete customization of input hyperparameters and selection of methods and offers a host of diagnostic plots and statistics to compare results between methods. Additionally, hadge performs joint genotype- and hashing-based deconvolution of cell mixtures generated from genetically diverse inputs to enable users to retrieve only confidently assigned singlets. This functionality is particularly relevant for experiments where hashing data quality may be compromised by technical noise, tissue-specific variability, or variability in reagent performance. In these experiments, genotype-free deconvolution followed by donor matching can increase the number of good-quality singlets which can be further investigated for biological signatures. Another recent work [18] proposed joint deconvolution to increase the confidence in called singlets, but offers limited options to customize the selection of tools or parameters to run the joint deconvolution step (Table 1). Given the importance of retaining only correctly assigned cells for downstream tasks, such as cell annotation and differential expression between multiplexed conditions, joint deconvolution is a necessary step for experiments threatened by suboptimal hashing libraries. Existing strategies generally only retain the union of singlets called by two methods [35]. Instead, hadge allows both automated matching of the best hashing and genotype-based deconvolution tools based on the optimal concordance between methods, or the selection of individual methods for each workflow, ensuring an additional level of control over the joint deconvolution step. To guarantee that the joint deconvolution retains only confidently donor-assigned singlets, we developed an additional refinement component that allows the generation of donor genotypes from recovered single-cell variants, which are then used as input for a new round of deconvolution. One limitation of this approach is that, by reducing the number of input variants to include only donor-specific variants, the read coverage in the already shallow-depth single-cell data may decrease at individual genetic variants, resulting in a higher number of cells discarded as negatives. Nevertheless, in the data presented here, only 15 (0.03%) of the total cells are misclassified into a different donor at this step, suggesting the relevance of the selected genetic variants. Furthermore, applying the joint demultiplexing approach can reduce the cost of multi-sample, multi-condition experiments, when the same donors are challenged with multiple perturbations. In such cases, staining only one condition provides enough data to generate donor-specific genotypes, removing the need for additional genotyping and reducing the costs of the staining procedure.

Other pipelines have been proposed to benchmark either genotype-based [23] or hashing-based deconvolution [16, 24] individually (Table 1). However, some deconvolution tools do not integrate well with downstream analysis pipelines, making it difficult to perform integrated analyses across multiple samples or experiments. hadge seamlessly integrates within the scverse [38] ecosystem, and its outputs can be processed with existing pipelines for automated single-cell analysis [39], minimizing the friction between preprocessing and data analysis steps and ensuring quality and reproducibility of results.

## Conclusions

In conclusion, hadge is a powerful and flexible pipeline that addresses the challenges associated with all commercially available single-cell multiplexing techniques in genomics studies. By allowing customization of input parameters, selection of methods, and joint deconvolution, hadge ensures confident identification of cell mixtures and retrieval of high-quality singlets. Its integration with existing analysis pipelines and compatibility with the scverse ecosystem further streamlines the data processing and analysis workflow, promoting reproducibility and enabling integrated analyses across multiple samples and experiments. With its comprehensive features and robust performance, hadge is poised to greatly enhance the accuracy and efficiency of single-cell genomics research.

## Methods

### Implementation of the hadge pipeline

The hadge pipeline, implemented in Nextflow, provides hashing- and genotype-based deconvolution workflows. Both workflows support the execution of multiple methods simultaneously.

#### Tools

The genotype-based deconvolution workflow includes five deconvolution methods: Demuxlet [17], Freemuxlet [26], Vireo [22], scSplit [20], and Souporcell [21].

The hashing-based deconvolution workflow includes seven hashing deconvolution algorithms: HTODemux [27], Multiseq [12], HashedDrops [28], Demuxem [15], gmm-demux [29], BFF [24], and Hashsolo [30], and one doublet-detection method (Solo [30]).

In addition to the two multiplexing workflows, the hadge pipeline includes a doublet detection method, Solo, which is based on a semi-supervised deep learning approach. Since Solo only identifies singlets without revealing the true donor identity of the droplets, we only use it as a supplementary method.

As genotype-based deconvolution techniques rely on SNPs to distinguish samples in the pools, the workflow also includes a preprocessing component with samtools, Freebayes [40] and cellsnp-lite [41] as two separate processes for variant calling. The Freebayes process is designed as per the instruction of scSplit (https://github.com/jon-xu/scSplit) to find variants in pooled samples. To optimize runtime, the process is carried out separately for each chromosome. With an additional filtering step, SNPs with a minimum allele count of 2, minimum base quality of 1 and quality scores greater than 30 from each chromosome are retained and merged. As suggested by Vireo, the Mode 1a of cellsnp-lite is called in the cellsnp-lite process to genotype single cells against candidate SNPs. Two allele count matrices for each given SNP are generated, one for the reference and another one for the alternative allele, which can be then fed into Vireo.

The hashing-based deconvolution workflow also has a pre-processing step to prepare the input data for both HTODemux and Multiseq based on the Seurat vignette (https://satijalab.org/seurat/articles/hashing_vignette.html). A Seurat object is initialized with the cell containing barcodes for the RNA matrix and HTO raw count matrix. Only the cell barcodes that are at the intersection between RNA and HTO counts are retained. The HTO data is added as an independent assay and normalized using centered log-ratio transformation (CLR).

#### Structure

The hadge pipeline features three distinct modes: *genetic, hashing*, and *rescue* mode. The hashing and genetic mode are two independent workflows, and the rescue mode allows joint demultiplexing by combining the outputs of the two workflows. Different inputs are required for the two workflows, specifically:

For the hashing workflow, raw and filtered HTO and RNA counts are the minimum required input. Each of these outputs is normally generated by the cellranger pipeline, which outputs the required HTO and RNA counts in the unfiltered (raw) and filtered feature-barcode matrices in two file formats: the Market Exchange Format (MEX), and Hierarchical Data Format (HDF5). Hadge accepts the files in the MEX format.

For the genetic workflow, the minimal requirements are as follows: the indexed sequence alignment file in BAM format along with its index (.BAI format), the barcodes of the cell-containing droplets in a TSV file, the number of expected donors in a mixture, and the reference genotypes and the variants present in the pooled sample, both in VCF format. The VCF of the reference genotype can be an unrelated genomic reference to run methods in “genotype-free” mode. Optionally, if the pooled sample’s VCF is not available, we include two processes for variant calling (cellsnp and freebayes). Users can provide the mixed FASTA file to be used as input to generate the VCF file with freebayes, which is the default preprocessing for the scSplit method. All of the inputs, except for the reference VCF files, are commonly generated by the cellranger pipeline. Following deconvolution in each workflow, the output files are passed to the summary process to generate summary files. Within this module, three CSV files are produced per tool as output, with each column representing a trial conducted during a single run of the pipeline. These output files provide a comprehensive summary of three aspects, including the specified parameters for each trial, the classification of individual droplets as singlets, doublets, or negative droplets, and the assignment of cell barcodes to their respective donors. As multiple tools are executed within a single run, additional CSV files are generated to merge the classification and assignment results from different tools into unified data frames.

In the rescue mode, hashing and genotype-based deconvolution workflows work jointly with the aim (i) to recover the droplets where the classification is discordant between the two approaches and (ii) optionally to extract donor-specific variants from the droplets with coherent classification and to reconstruct donor genotypes for mixed samples, which can then be used to rerun genotyped-based deconvolution as a sanity check to prove whether the result is reliable. The pipeline first runs the two workflows in parallel and saves the results of all methods in a single CSV file. Next, the file is passed to the “donor matching” process which computes a score (Phi score) to associate an identity to the anonymous donors using the droplets where the concordance between one genetic donor and one hashtag is maximized.

The process converts the assignment of two tools into a matrix of binary values, with rows representing cell barcodes and columns representing donors or hashtags. The value is set to 1 if the cell is assigned to the donor or hashtag, and 0 otherwise. The similarity between two matrices is calculated column-by-column using Pearson correlation, and hashtags and donors are matched if they have the highest mutual Pearson correlations. If every donor is paired with a hashtag, the pipeline generates a new assignment of the tools with mapped donors and a heat map to visualize the correlation between the donors and hashtags. If Vireo is the optimal genotype-based deconvolution method in donor matching, the process has the option to extract informative variants from donor genotypes estimated by Vireo. Using the input of cellsnp-lite, genotyped SNPs are first filtered based on the SNPs (read depth > 10) among cells with consistent assignment between Vireo and the hashing tool with which it is compared. Only variants with an overrepresented allele are retained, i.e., the frequency of the alternative or reference allele in the group of cells must be greater than a specified threshold (frequency > 90%). The pipeline compares the genotypes of these variants in cells that have been inconsistently deconvolved and keeps only the SNPs that have the same overrepresented allele in cells with and without consistent assignment. These are candidate variants used to distinguish cells from different donors. The process is performed separately on cells from different donors to retrieve donor-specific informative variants. Finally, BCFtools sorts and indexes the donor genotype from Vireo and filters the donor-specific variants. The samples are renamed by the matching hashtags.

#### Demuxlet/Freemuxlet

Dsc-pileup, Demuxlet, and Freemuxlet implemented in popscle (v0.1) were performed one after another. Using the BAM file and filtered barcode file produced by cellranger [42] as input, dsc-pileup aggregated reads around common SNPs in the human population, which in the case of Freemuxlet are derived from the 1000 Genomes Project and filtered by cellsnp-lite with minor allele frequency (MAF) > 0.05 as reference variant sites (https://sourceforge.net/projects/cellsnp/files/SNPlist/). Demuxlet/Freemuxlet then uses the pileup files from dsc-pileup to deconvolve cells. We ran these methods in default mode.

#### Vireo

Cell genotypes were generated at common SNPs from the 1000 Genomes Project (https://sourceforge.net/projects/cellsnp/files/SNPlist/) using cellsnp-lite (v1.2.2) with default parameters before performing Vireo. Subsequently, the output of cellsnp-lite was processed by Vireosnp (v0.5.6) to perform the deconvolution with default parameters.

#### Souporcell

Souporcell (v2.0) was run on the BAM file and filtered barcode file produced by cellranger and the human reference (http://cf.10xgenomics.com/supp/cell-exp/refdata-cellranger-GRCh38-3.0.0.tar.gz). We also used common SNPs from the 1000 Genomes Project [43] with a minor allele frequency of 2% (provided by https://github.com/wheaton5/souporcell) as input to skip repeated and memory-intensive steps, remapping, and variant-calling.

#### scSplit

scSplit was executed only after the pre-processing and variant calling modules were completed. The input BAM file was pre-processed by SAMtools (v1.15.1) and UMI-tools (v1.1.2). In the variant calling module, freebayes (v1.2) was performed on the pre-processed BAM file to call variants from mixed samples. Taking the pre-processed BAM file and called variants, scSplit (v1.0.8.2) deconvolved the cell mixture in three steps. The count command of scSplit constructed two count matrices for the reference and alternative alleles. To increase the accuracy of donor identification, a list of common SNPs provided by scSplit (https://melbourne.figshare.com/articles/dataset/Common_SNVS_hg38/17032163) was used to filter the count matrices. The run command identified the cells in the pool to the clusters according to the allele matrices, with doublets being assigned to a separate cluster. Finally, the genotype command predicted individual genotypes for every cluster.

#### HTODemux

HTODemux begins with loading the Seurat object, which was created during the pre-processing module using the Seurat R package (v4.3.0). HTODemux (also included in Seurat R package v4.3.0) was then called with default parameters to deconvolve cells based on clr-normalized HTO counts.

#### Multiseq

Similar to HTODemux, MULTIseqDemux (included in Seurat R package v4.3.0) function was performed on the pre-processed Seurat object, with default parameters allowing for automatic determination of the optimal quantile to use in a range from 0.1 to 0.9 by a step of 0.05.

#### Demuxem

We used Demuxem with default settings. The raw RNA and HTO count data were loaded as a MultimodalData object (pegasuspy Python package v1.7.1). Demuxem then deconvolved cells with at least 100 expressed genes and 100 UMIs in two main steps. The antibody background was first determined based on empty barcodes using the KMeans algorithm. The signal hashtag counts were then calculated using background information, and cells with a minimum signal of 10 were assigned to their signal hashtag.

#### Hashsolo

The process expects to start from the raw HTO counts in hdf5 file format into an Anndata object (Scanpy v1.9.1) (solo-sc v1.3). We ran Hashsolo with default parameters, setting the priors for the hypothesis of negative droplets, singlets, and doublets each to 1/3.

#### HashedDrops

This process requires as input both RNA and HTO raw counts. First, emptyDrops finds cell-containing droplets, this list of barcodes is then used as input to the HashedDrops call (both algorithms are included in DropletUtils R package v1.18.0). We used HashedDrops with default settings.

#### BFF

BFF accepts raw or preprocessed HTO data, while offering a preprocessing step (ProcessCountMatrix), included in the CellHashR pipeline (CellHashR v.1.14.0). Two different alternatives of BFF are available, “BFF raw” and “BFF cluster,” which apply different processing on the HTO raw counts. Both methods can be run in parallel and the tool will generate a consensus call between the two. We ran both alternatives for the benchmark.

#### Gmm-demux

GMM-demux (GMM-demux Python package v.0.2.1.3) expects the HTO raw counts as csv or tsv files and the names of the expected cell hashtags. We ran GMM-demux using tsv files under default parameters.

#### Benchmarking

## mpxMS-dataset

We were granted early access to a dataset generated in a study of progressive multiple sclerosis (Calliope Dendrou, University of Oxford) [35]. In brief, this dataset includes a multiplexed 3′ single nuclear RNA sequencing dataset of brain tissue from 5 controls and 5 cases of progressive multiple sclerosis (mpxMSdataset). The mpxMS-dataset is divided into two sequencing batches (gx12 and gx38) of 6 donors each, with the individual donors hashed with one of six unique TotalSeq™-A anti-nuclear pore complex antibodies. We obtained the count data generated with Cellranger v3.1.0: 6,794,833 barcodes and 6,794,880 barcodes were detected in the raw data of gx12 and gx38, respectively. The number of cells detected in each experiment before deconvolution was 4889 for gx12 and 13,184 for gx38.

The pipeline was applied to the mpxMS-dataset in the rescue mode. In the genotyped-based deconvolution workflow, Freemuxlet, Vireo, Souporcell, and scSplit were used in the absence of reference donor genotypes. To run the algorithm, the number of samples was set to six. All hashing-based deconvolution methods were called to deconvolute the data. All output files were gathered and passed to the corresponding summary component (R v4.2.2). The results of Vireo and Multiseq were used to map donor identities to hashtags in the donor matching component. Donor genotypes estimated by Vireo were then processed by BCFtools (v1.8). The donor-specific variants were extracted from the donor genotypes, where the cell variants were filtered by a minimal cell count of 10 and the overrepresented allele at a given SNP was then determined by a 90% cut-off.

Data analysis was performed with scanpy (v1.9.3) and scrublet (v0.2.3). Plots were generated with scanpy (v1.9.3), seaborn (v0.12.2), and matplotlib (v3.7.1).

We generally followed the recommendations given by the developers of the package (https://scanpy.readthedocs.io/en/stable/index.html) and have in part adjusted for this dataset and in accordance with analysis best practices [44].

For analysis, log-transformation and normalization were achieved with scanpy’s log1p() and normalize_total() function. After this, 50 PCs were generated by principal component analysis (PCA) and dimensionality reduction by UMAP was performed using scanpy’s pca() and umap() functions respectively. Cluster identification was performed using the Leiden algorithm and differential expression of the different clusters was generated using scanpy’s rank_genes_groups() function.

## OneK1k-dataset

Raw oneK1K data for scRNA-seq and microarray-based genotype were retrieved from the GEO database (accession numbers GSE196735, GSE196829). Ground truth cell barcode assignment was extracted from the deposited single-cell data (https://cellxgene.cziscience.com/collections/dde06e0f-ab3b-46be-96a2-a8082383c4a1). The demultiplexing was carried out by specifying the original number of donors in each pool (https://www.ncbi.nlm.nih.gov/geo/query/acc.cgi?acc=GSE196830) and using the donor-genotypes VCFs extracted from the pools bam files using cellsnp, matched against the whole population genotypes. Since the deposited single-cell data contains less cells and donors than the full demultiplexing results (981 as opposed to 1015 donors), the analysis on the percent matched donors was carried out on the 981 donors present in the demultiplexing results. Genotype imputation was performed using the TOPMED-r2 Minimac4 1.7.4 imputation tool. For scRNA-seq data, alignment was conducted using Cellranger version 6.1.1. Hadge genetic demultiplexing was applied under default settings in both genotype aware and genotype absent modes and to ensure tool comparability and data consistency; default cellsnp common variants were used. (https://sourceforge.net/projects/cellsnp/files/SNPlist/).

## Cell number downsampling experiments

We performed consecutive downsampling of two publicly available hashing datasets, a PBMC multiplexed sample with 4 hashtags and a total of 15,843 cells (https://www.ncbi.nlm.nih.gov/geo/query/acc.cgi?acc=GSE152981) and the test data used in the cellHashR pipeline, hereby called CR-438–21, for a total of 11,090 cells (https://www.github.com/BimberLab/cellhashR/tree/master/tests/testdata/438-21-GEX) which also provides raw rna data to enable running demuxem. The same dataset was also used for the run time benchmarks (see “Run time benchmarks”). For each sample, we applied random downsampling to 30–50-70% of the cell barcodes in the HTO matrix, using five different seeds to control for performance variations. The downsampled counts were then fed to the hadge hashing workflow and each method was run under default conditions. For the PBMC dataset, the ground truth labels were obtained running gmm-demux on the full sample as described in the original publication [29]. For the CR-438–21, the ground truth labels were obtained using the instructions provided on the cellhashR repository. The scripts used for downsampling are available at https://github.com/theislab/hadge-reproducibility.

Downsampling of the genetic data was performed on the first batch (gx12) of mpxMS-dataset, using as ground truth the joint deconvolution results between vireo and multiseq demonstrated in Figs. 3 and 4. We performed random downsampling to 30% and 50% of the cell barcodes associated with each donor, using three different seeds, and evaluated the percentage matching singlets recovered after removing 30 or 50% of the cells for a particular donor across the different tested seeds. To reduce the run time, bam files were pre-processed to contain only reads that overlap with known common SNPs from the 1000 Genomes Project (https://sourceforge.net/projects/cellsnp/files/SNPlist/). The downsampled reads were then used as input for hadge genetic workflow and each method was run under default conditions.

## Run time benchmarks

We benchmarked the performance of hadge against demuxafy and cellhashR using four samples with different cell numbers. Each pipeline is developed in different frameworks and requires different configurations. In the demuxafy pipeline, genotype-based deconvolution methods were called sequentially within the singularity container. The benchmark was run on the mpxMS-dataset batch gx12 and gx38, the cellhashR dataset as well as a reduced test dataset provided by demuxafy, using the same parameters as hadge. Since demuxafy does not provide preprocessing functions, we used hadge’s preprocessing module ( which includes *freebayes*, *samtools*, and *cellsnp*) to provide the same input data to hadge-genetic and demuxafy so the benchmarking starts from the same inputs. For the cellhashR pipeline, we created a conda environment with all the required dependencies as described in the cellhashr GitHub repository [45]. In the hadge-hashing pipeline, each deconvolution method was called in its own Conda environment separately. For each pipeline run, we allowed 160-GB RAM memory and 32 CPU cores.

### Supplementary Information

**Additional file 1:** Supplementary figures.

**Additional file 2:** Supplementary table containing the donor assignments per dataset and tool.

**Additional file 3:** Supplementary tables.

**Additional file 4:** Review History.

## Data Availability

The hadge source code is available at https://github.com/theislab/hadge [46] under the MIT license. We also deposited the version that we used for this manuscript to Zenodo [47]. Further documentation, tutorials and examples are available at https://hadge.readthedocs.io/en/latest. Jupyter notebooks to reproduce our analysis and figures including Conda environments that specify all versions are available at https://github.com/theislab/hadge-reproducibility and also deposited to Zenodo [47]. The mpsMS-dataset applied in this study is an unpublished dataset obtained directly from the authors [35]. The onek1k data is publicly accessible via the National Center for Biotechnology Information (NCBI) Sequence Read Archive (SRA) under accession number SRX14182577 [48]. The PBMC hashing dataset is publicly available on GEO with accession number GSE152981 [49]. The CR-438–21 sample is publicly available (https://www.github.com/BimberLab/cellhashR/tree/master/tests/testdata/438-21-GEX).
