# Supplementary material for: hadge: a comprehensive pipeline for donor deconvolution in single-cell studies
Source: Genome Biol. 2024 Apr 26;25:109. doi: 10.1186/s13059-024-03249-z (PMC11055383; doi:10.1186/s13059-024-03249-z)
Supplement: Supplementary file 1 — Additional file 1: Supplementary figures. [file 13059_2024_3249_MOESM1_ESM.pdf]

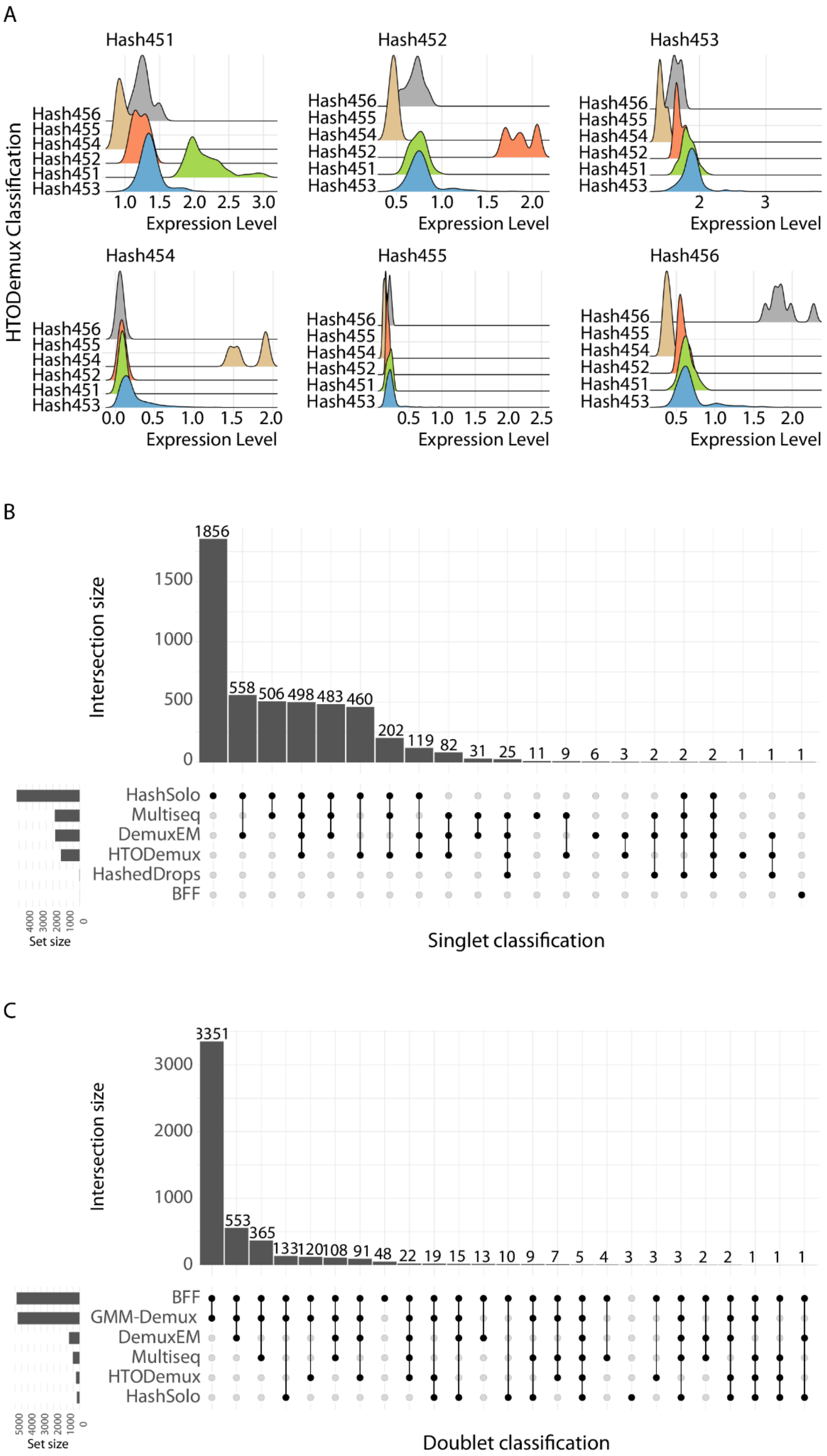
A
HTODemux Classification
Hash451
Hash452
Hash453
Hash454
Hash455
Hash456
Expression Level
B
Intersection size
1856
558
506
498
483
460
202
119
82
31
25
11
9
6
3
2
2
2
1
1
1
HashSolo
Multiseq
DemuxEM
HTODemux
HashedDrops
BFF
Set size
Singlet classification
C
Intersection size
3351
553
365
133
120
108
91
48
22
19
15
13
10
9
7
5
4
3
3
3
2
2
1
1
1
BFF
GMM-Demux
DemuxEM
Multiseq
HTODemux
HashSolo
Set size
Doublet classification

Fig. S1. Different performance of hashing-based deconvolution methods. (A) Ridge plot of HTO expression level grouped by cells assigned to different hashtags indicates a relatively similar expression level of different HTOs in cells of Hashtag 454 and 455. (B) Upset plot representing different singlet classification by hashing-based deconvolution methods. Horizontal bars represent the total number of singlets classified by each method. The vertical bars depict the overlapping singlet classifications, indicated by black circles, where singlets are classified by a single method or a combination of methods. (C) Upset plot representing different doublet classification by hashing-based deconvolution methods.

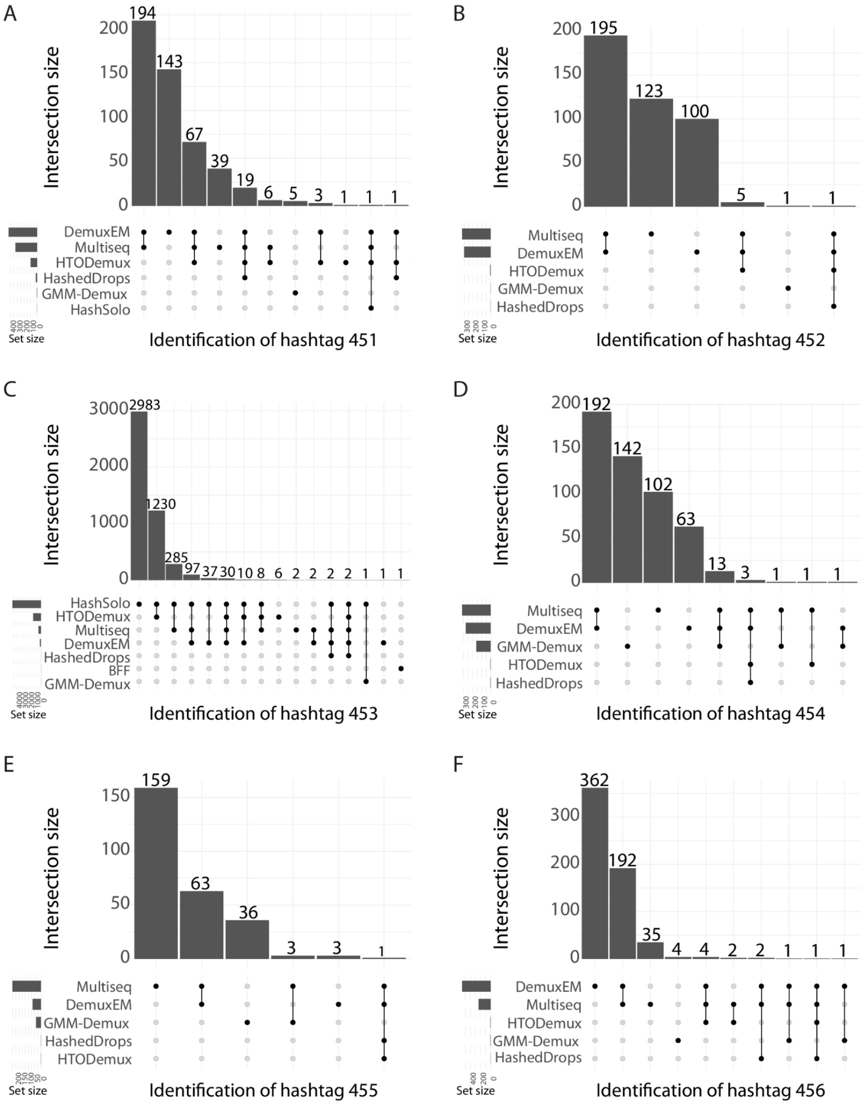

Fig. S2. Discordant donor identification performance of hashing-based deconvolution methods on mpxMS-dataset batch gx12.(A) - (E) Upset plot representing different donor assignment of hashing-based deconvolution methods for (A) Hashtag 451, (B) Hashtag 452, (C) Hashtag 453, (D) Hashtag 454, (E) Hashtag 455 and (F) Hashtag 456. Horizontal bars represent the total number of cells assigned to the hashtag by each method. The vertical bars depict the overlapping donor assignment, indicated by black circles, where cells are assigned by a single method or a combination of methods.

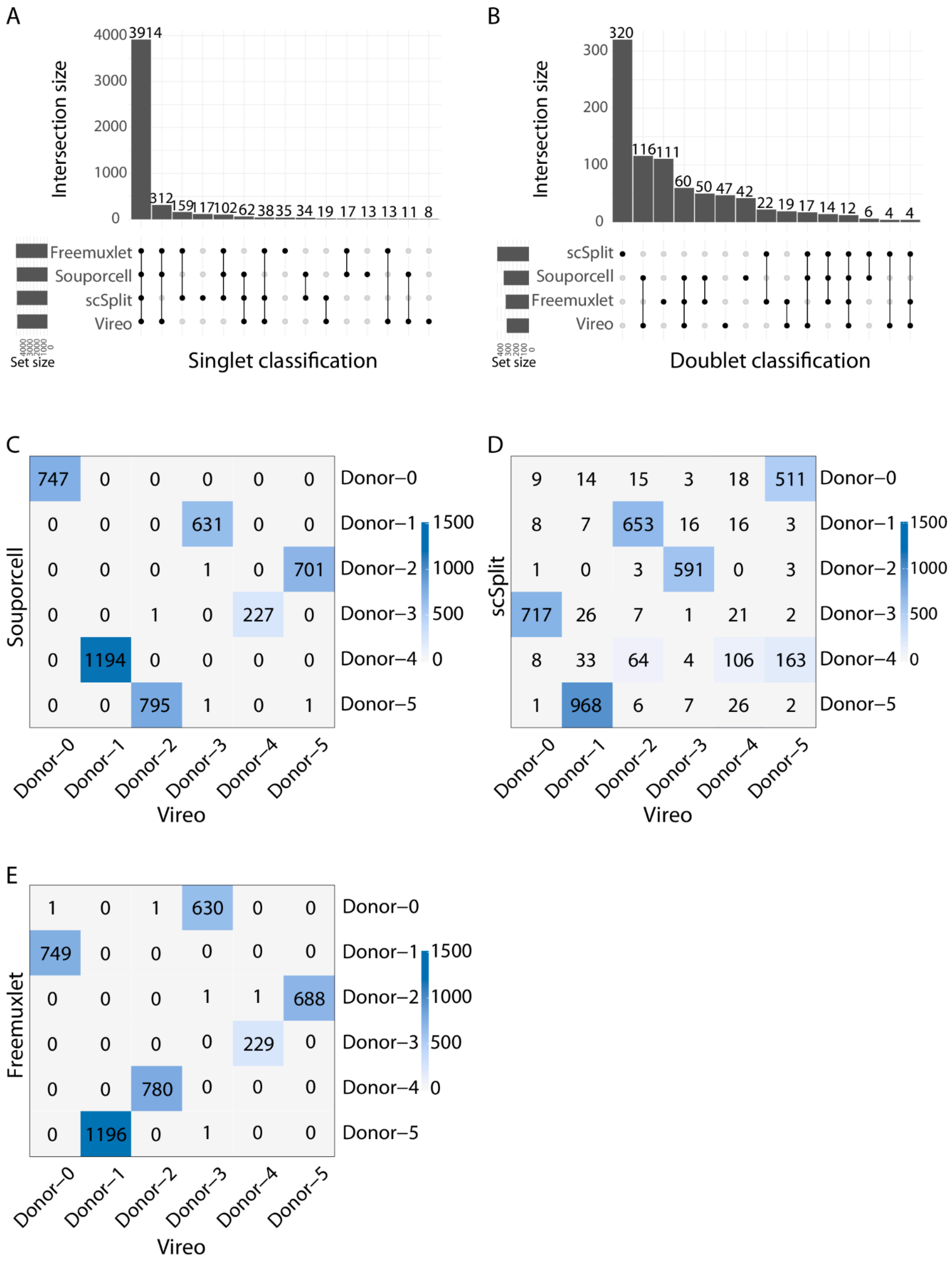

Fig. S3. Concordant donor identification performance of genotype-based deconvolution methods on mpxMS-dataset batch gx12. (A) Upset plot representing different singlet classification by genotype-based deconvolution methods. Horizontal bars represent the total number of singlets classified by each method. The vertical bars depict the overlapping singlet classifications, indicated by black circles, where singlets are classified by a single method or a combination of methods. (B) Upset plot representing different doublet classification by genotype-based deconvolution methods. (C) - (E) Confusion matrix representing the agreement in donor identification between Vireo and other three methods: (C) Souporcell, (D) scSplit and (E) Freemuxlet. Vireo is fixed on the x-axis as baseline. The rows and columns represent the anonymous donor clusters genotyped by each respective method. The values within the cells represent the number of singlets assigned to each specific donor cluster.

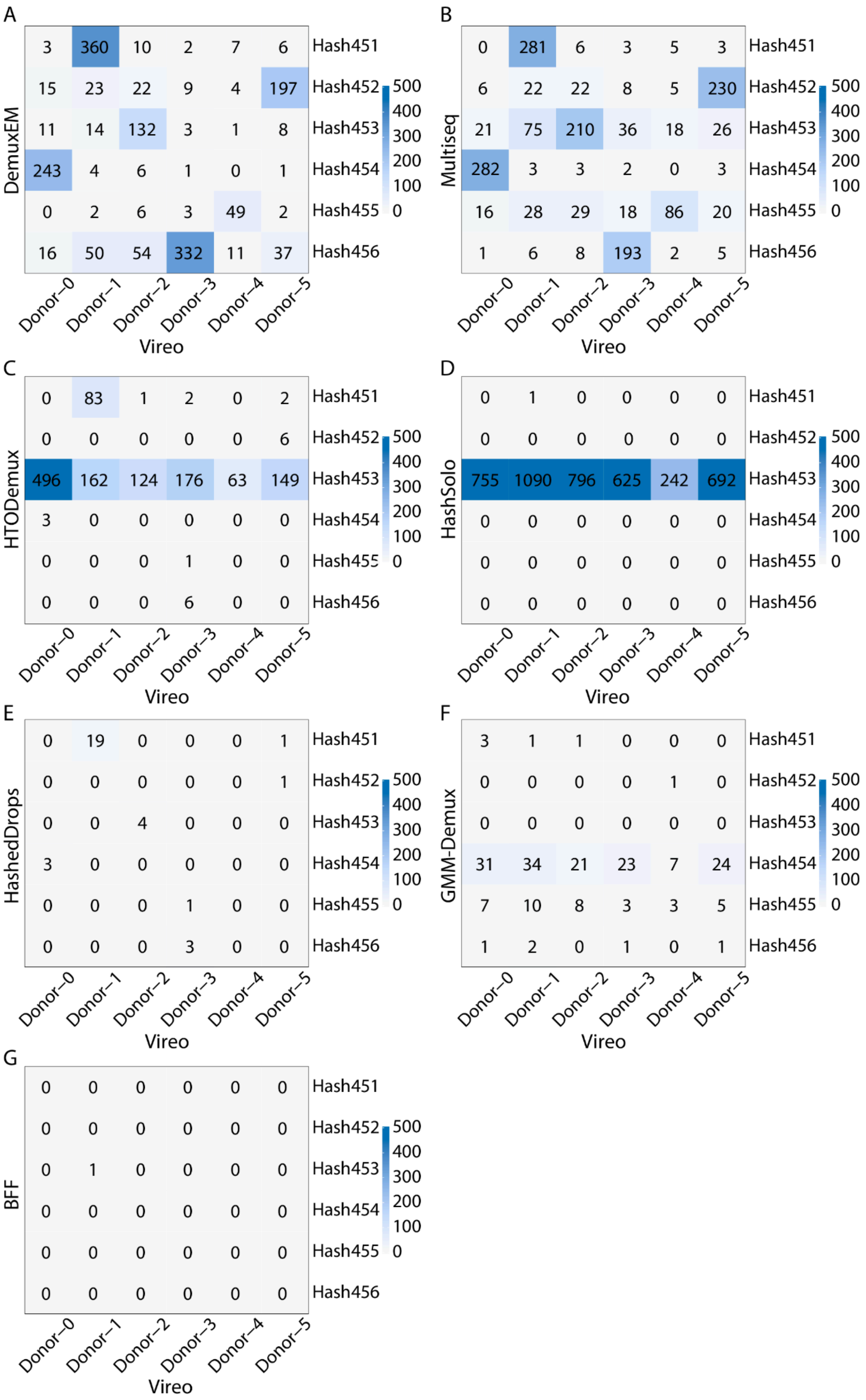
A
DemuxEM
3 360 10 2 7 6 Hash451
15 23 22 9 4 197 Hash452
11 14 132 3 1 8 Hash453
243 4 6 1 0 1 Hash454
0 2 6 3 49 2 Hash455
16 50 54 332 11 37 Hash456
Donor–0 Donor–1 Donor–2 Donor–3 Donor–4 Donor–5
Vireo
500 400 300 200 100 0
B
Multiseq
0 281 6 3 5 3 Hash451
6 22 22 8 5 230 Hash452
21 75 210 36 18 26 Hash453
282 3 3 2 0 3 Hash454
16 28 29 18 86 20 Hash455
1 6 8 193 2 5 Hash456
Donor–0 Donor–1 Donor–2 Donor–3 Donor–4 Donor–5
Vireo
500 400 300 200 100 0
C
HTODemux
0 83 1 2 0 2 Hash451
0 0 0 0 0 6 Hash452
496 162 124 176 63 149 Hash453
3 0 0 0 0 0 Hash454
0 0 0 1 0 0 Hash455
0 0 0 6 0 0 Hash456
Donor–0 Donor–1 Donor–2 Donor–3 Donor–4 Donor–5
Vireo
500 400 300 200 100 0
D
HashSolo
0 1 0 0 0 0 Hash451
0 0 0 0 0 0 Hash452
755 1090 796 625 242 692 Hash453
0 0 0 0 0 0 Hash454
0 0 0 0 0 0 Hash455
0 0 0 0 0 0 Hash456
Donor–0 Donor–1 Donor–2 Donor–3 Donor–4 Donor–5
Vireo
500 400 300 200 100 0
E
HashedDrops
0 19 0 0 0 1 Hash451
0 0 0 0 0 1 Hash452
0 0 4 0 0 0 Hash453
3 0 0 0 0 0 Hash454
0 0 0 1 0 0 Hash455
0 0 0 3 0 0 Hash456
Donor–0 Donor–1 Donor–2 Donor–3 Donor–4 Donor–5
Vireo
500 400 300 200 100 0
F
GMM-Demux
3 1 1 0 0 0 Hash451
0 0 0 0 1 0 Hash452
0 0 0 0 0 0 Hash453
31 34 21 23 7 24 Hash454
7 10 8 3 3 5 Hash455
1 2 0 1 0 1 Hash456
Donor–0 Donor–1 Donor–2 Donor–3 Donor–4 Donor–5
Vireo
500 400 300 200 100 0
G
BFF
0 0 0 0 0 0 Hash451
0 0 0 0 0 0 Hash452
0 1 0 0 0 0 Hash453
0 0 0 0 0 0 Hash454
0 0 0 0 0 0 Hash455
0 0 0 0 0 0 Hash456
Donor–0 Donor–1 Donor–2 Donor–3 Donor–4 Donor–5
Vireo
500 400 300 200 100 0

Fig. S4. Different donor identification performance between hashing and genotyped-based deconvolution methods on mpxMS-dataset batch gx12. (A) - (G) Confusion matrix representing the agreement in donor identification between Vireo and other five hashing-based deconvolution methods: (A) Demuxem, (B) Multiseq, (C) HTODemux, (D) HashSolo, (E) HashedDrops, (F) GMM-Demux and (G) BFF. Vireo is fixed on the x-axis as baseline. The rows represent the hashtags, while the columns represent the anonymous donor clusters genotyped by Vireo. The values within the cells represent the number of singlets assigned to each specific donor or hashtag.

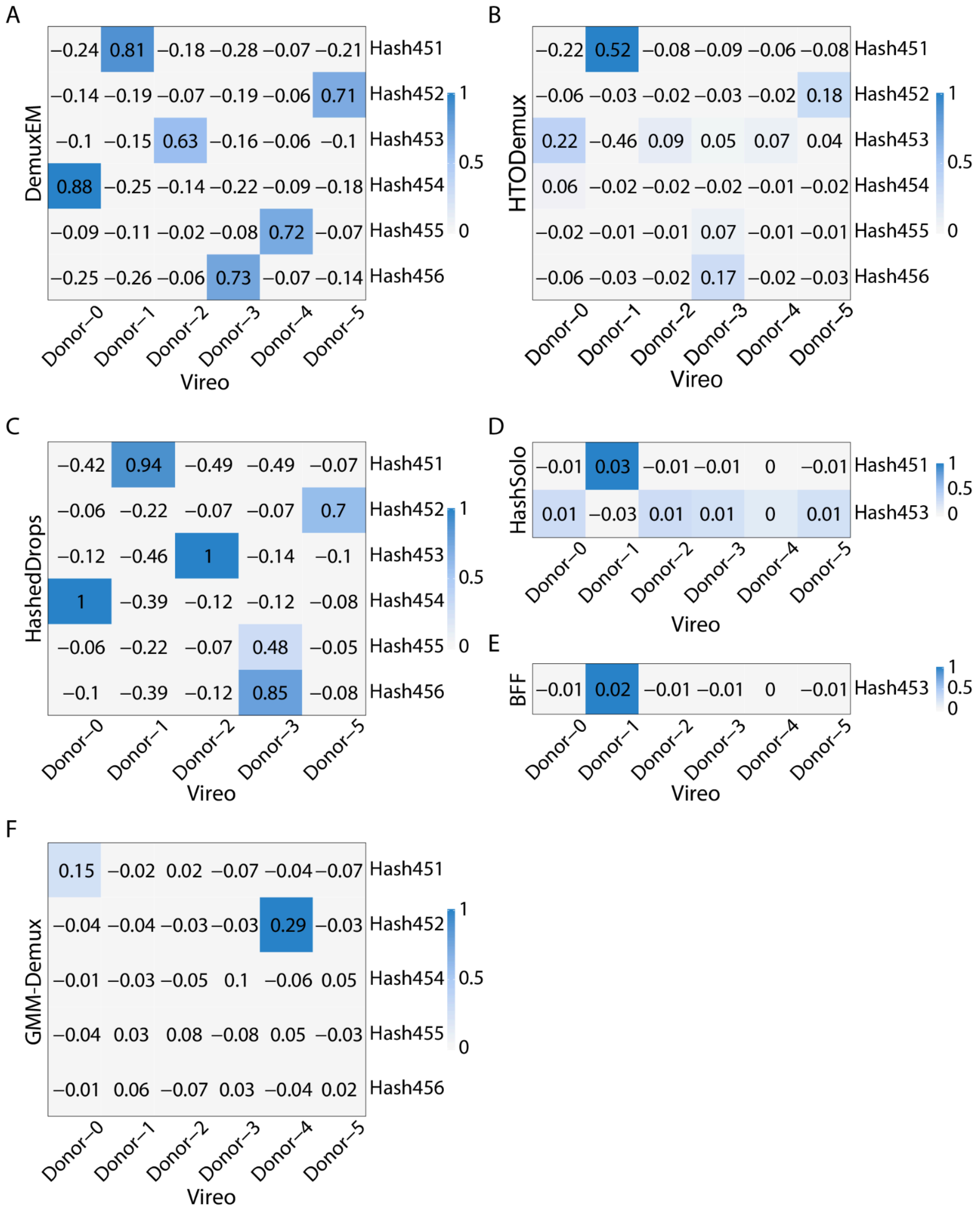

Fig. S5. Different donor identification performance between hashing and genotyped-based deconvolution methods on mpxMS-dataset batch gx12 based on Pearson correlation. (A) - (E) Correlation matrix representing the agreement in donor identification between Vireo and other five hashing-based deconvolution methods: (A) Demuxem, (B) HTODemux, (C) HashedDrops, (D) HashSolo, (E) BFF and (F) GMM-Demux. Vireo is fixed on the x-axis as baseline. The rows represent the hashtags, while the columns represent the anonymous donor clusters genotyped by Vireo. The values within the cells represent the Pearson correlation score between singlets assigned to a specific hashtag and those assigned to a donor cluster.

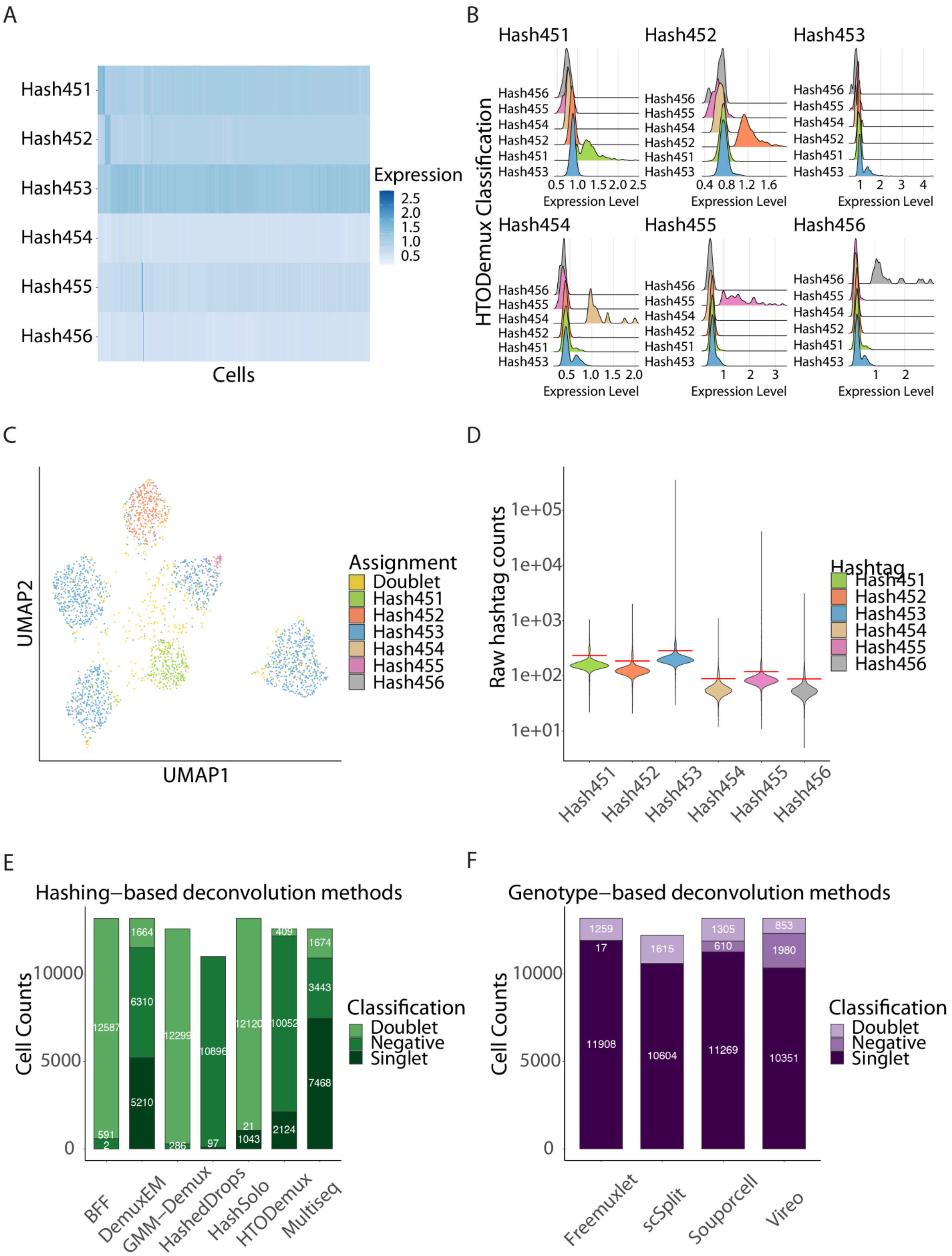

Fig. S6. Comparison of the performance of donor deconvolution methods on mpxMS-dataset batch gx38. (A) The heatmap of normalised HTO counts per cell is dominated by Hashtag 453 with noisy or undetectable expression of the other HTOs. (B) Ridge plot of HTO expression level grouped by cells assigned to different hashtags indicates a relatively similar expression level of different HTOs in cells of Hashtag 451 and 453. (C) t-SNE plot of normalised HTO counts coloured by HTODemux assignment shows poor separation of the cells based on hashtags, with most droplets assigned to Hashtag 453. (D) The violin plot of raw HTO counts shows a high count of Hashtag 453 in cells compared with the expression of the other HTOs. The (E) Bar plot shows the inconsistent classification of cells by hashing-based deconvolution methods. (F) Bar plot showing a more consistent assignment of the cell mixture to singlets, doublets and negatives by genotype-based deconvolution method.

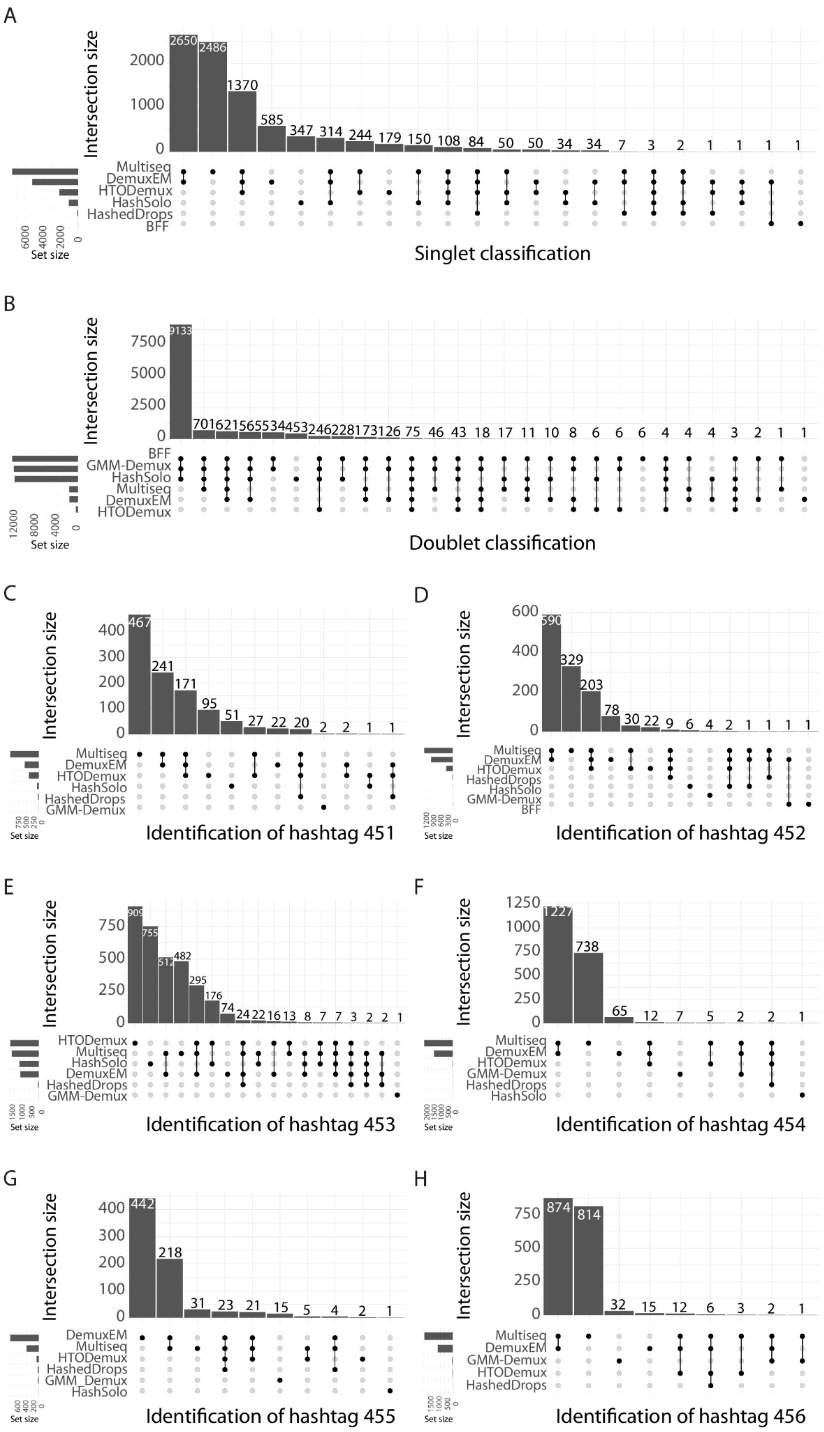

Fig. S7. Discordant performance of hashing-based deconvolution methods on mpxMS-dataset batch gx38. (A) Upset plot representing different singlet classification by hashing-based deconvolution methods. Horizontal bars represent the total number of singlets classified by each method. The vertical bars depict the overlapping singlet classifications, indicated by black circles, where singlets are classified by a single method or a combination of methods. (B) Upset plot representing different doublet classification by hashing-based deconvolution methods. (C) - (H) Upset plot representing different donor identification assignment by hashing-based deconvolution methods for (C) Hashtag 451, (D)Hashtag 452, (E) Hashtag 453, (F) Hashtag 454, (G) Hashtag 455 and (H) Hashtag 456.

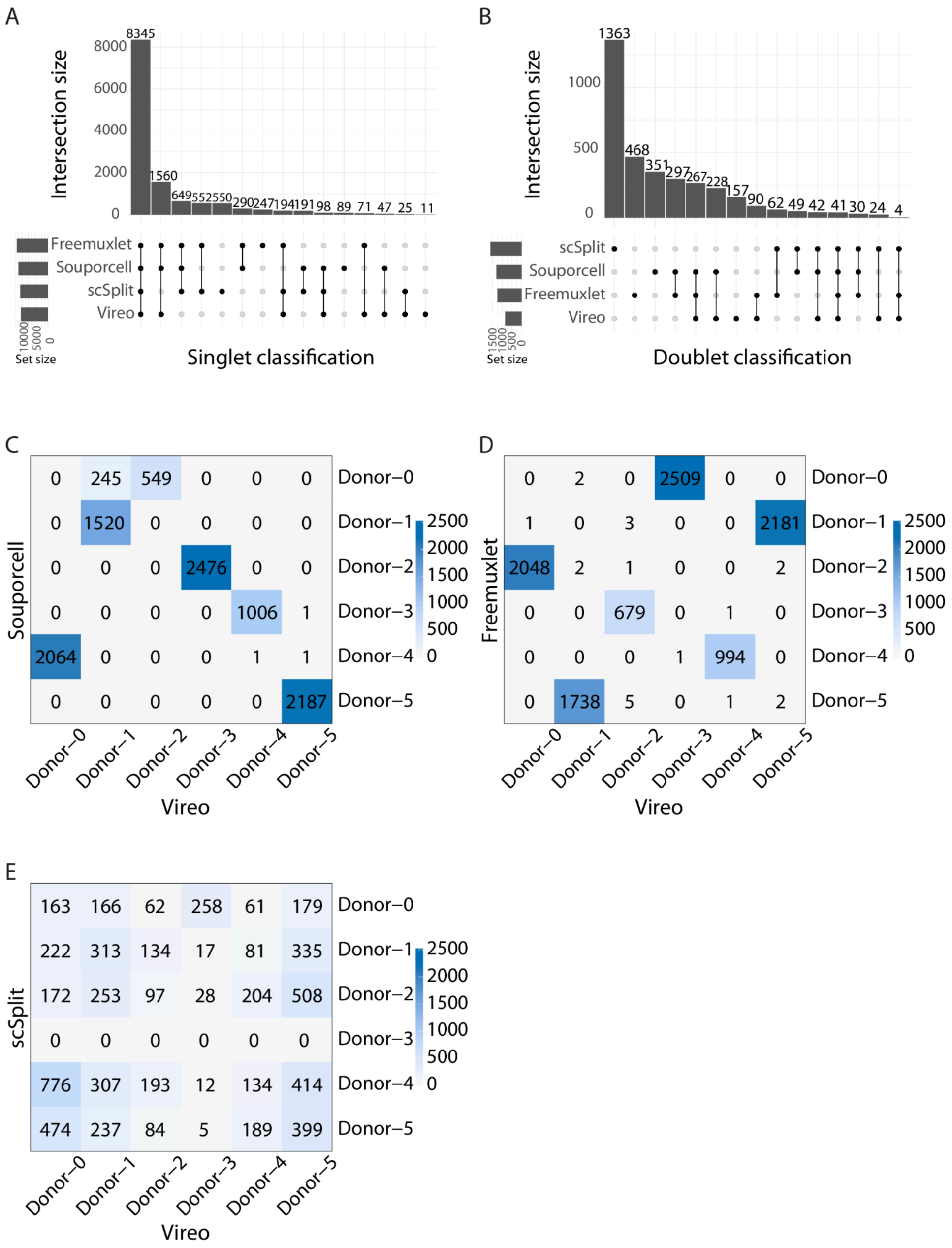

Fig. S8. Concordant performance among genotype-based deconvolution methods on mpxMS-dataset batch gx38. (A) Upset plot representing different singlet classification by genotype-based deconvolution methods. Horizontal bars represent the total number of singlets classified by each method. The vertical bars depict the overlapping singlet classifications, indicated by black circles, where singlets are classified by a single method or a combination of methods. (B) Upset plot representing different doublet classification by genotype-based deconvolution methods. (C)-(E) Confusion matrix representing the agreement in donor identification between Vireo and other three methods: (C) Souporcell, (D) Freemuxlet and (E) scSplit. Vireo is fixed on the x-axis as baseline. The rows and columns represent the anonymous donor clusters genotyped by each respective method. The values within the cells represent the number of singlets assigned to each specific donor cluster.

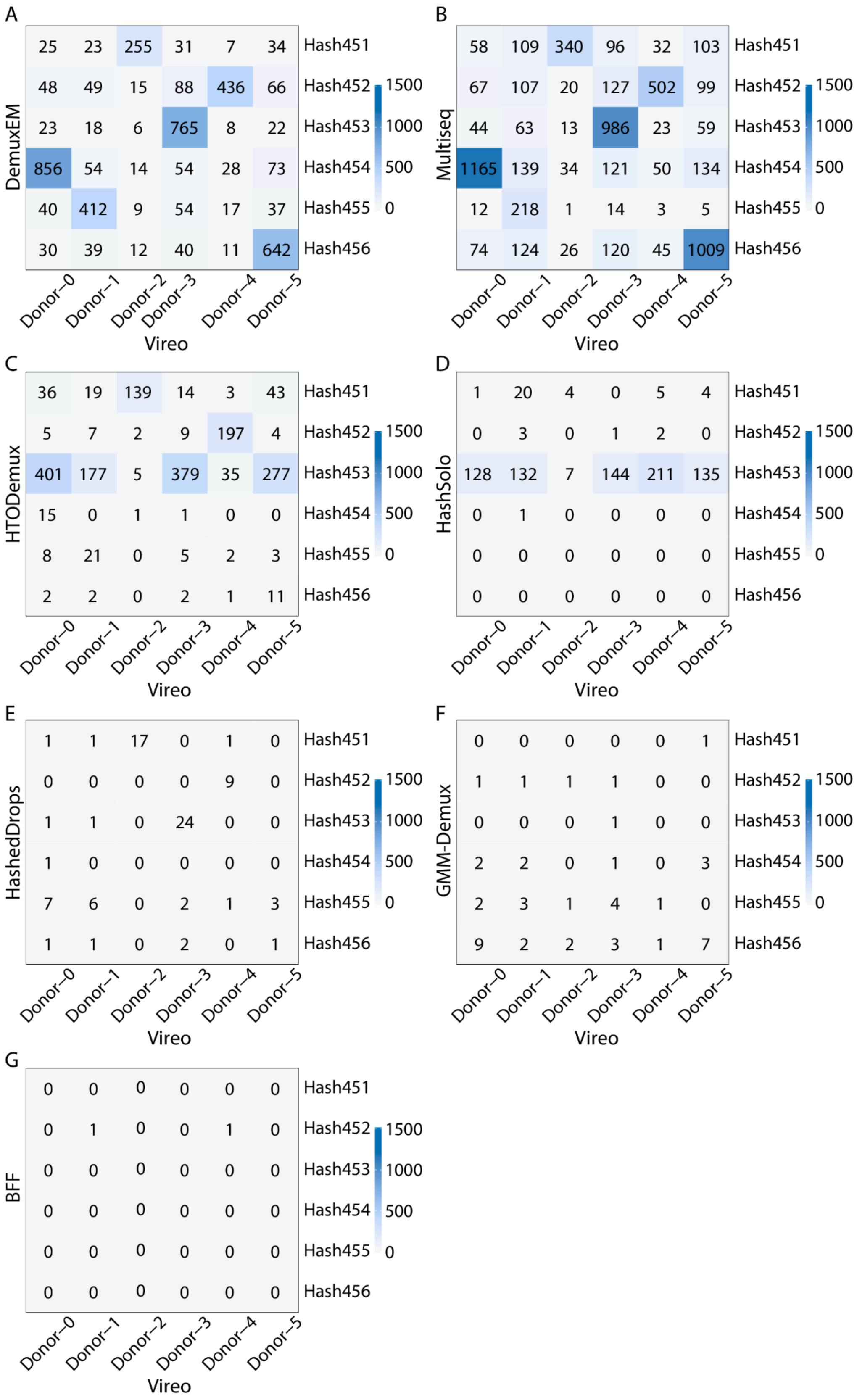

A
DemuxEM
25 23 255 31 7 34 Hash451
48 49 15 88 436 66 Hash452
23 18 6 765 8 22 Hash453
856 54 14 54 28 73 Hash454
40 412 9 54 17 37 Hash455
30 39 12 40 11 642 Hash456
1500 1000 500 0
Donor–0 Donor–1 Donor–2 Donor–3 Donor–4 Donor–5
Vireo
B
Multiseq
58 109 340 96 32 103 Hash451
67 107 20 127 502 99 Hash452
44 63 13 986 23 59 Hash453
1165 139 34 121 50 134 Hash454
12 218 1 14 3 5 Hash455
74 124 26 120 45 1009 Hash456
1500 1000 500 0
Donor–0 Donor–1 Donor–2 Donor–3 Donor–4 Donor–5
Vireo
C
HTODemux
36 19 139 14 3 43 Hash451
5 7 2 9 197 4 Hash452
401 177 5 379 35 277 Hash453
15 0 1 1 0 0 Hash454
8 21 0 5 2 3 Hash455
2 2 0 2 1 11 Hash456
1500 1000 500 0
Donor–0 Donor–1 Donor–2 Donor–3 Donor–4 Donor–5
Vireo
D
HashSolo
1 20 4 0 5 4 Hash451
0 3 0 1 2 0 Hash452
128 132 7 144 211 135 Hash453
0 1 0 0 0 0 Hash454
0 0 0 0 0 0 Hash455
0 0 0 0 0 0 Hash456
1500 1000 500 0
Donor–0 Donor–1 Donor–2 Donor–3 Donor–4 Donor–5
Vireo
E
HashedDrops
1 1 17 0 1 0 Hash451
0 0 0 0 9 0 Hash452
1 1 0 24 0 0 Hash453
1 0 0 0 0 0 Hash454
7 6 0 2 1 3 Hash455
1 1 0 2 0 1 Hash456
1500 1000 500 0
Donor–0 Donor–1 Donor–2 Donor–3 Donor–4 Donor–5
Vireo
F
GMM-Demux
0 0 0 0 0 1 Hash451
1 1 1 1 0 0 Hash452
0 0 0 1 0 0 Hash453
2 2 0 1 0 3 Hash454
2 3 1 4 1 0 Hash455
9 2 2 3 1 7 Hash456
1500 1000 500 0
Donor–0 Donor–1 Donor–2 Donor–3 Donor–4 Donor–5
Vireo
G
BFF
0 0 0 0 0 0 Hash451
0 1 0 0 1 0 Hash452
0 0 0 0 0 0 Hash453
0 0 0 0 0 0 Hash454
0 0 0 0 0 0 Hash455
0 0 0 0 0 0 Hash456
1500 1000 500 0
Donor–0 Donor–1 Donor–2 Donor–3 Donor–4 Donor–5
Vireo

Fig. S9. Different donor identification performance between hashing and genotyped-based deconvolution methods on mpxMS-dataset batch gx38. (A) - (G) Confusion matrix representing the agreement in donor identification between Vireo and other five hashing-based deconvolution methods: (A) Demuxem, (B) Multiseq, (C) HTODemux, (D) HashSolo, (E) HashedDrops, (F) GMM-Demux and (G) BFF. Vireo is fixed on the x-axis as baseline. The rows represent the hashtags, while the columns represent the anonymous donor clusters genotyped by Vireo. The values within the cells represent the number of singlets assigned to each specific donor or hashtag.

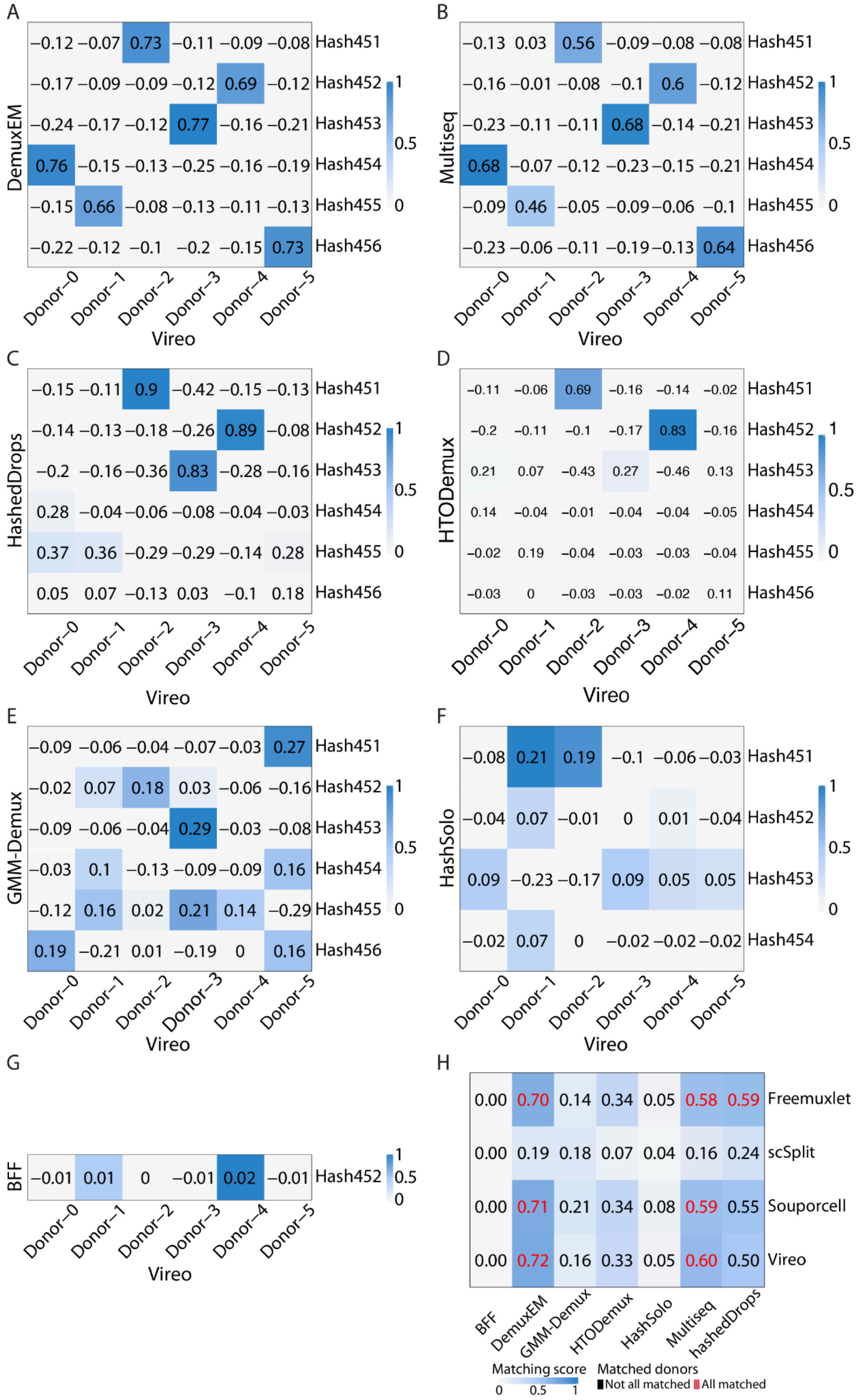

Fig. S10. Different donor identification performance between hashing and genotyped-based deconvolution methods on mpxMS-dataset batch gx38. (A) - (G) Correlation matrix representing the agreement in donor identification between Vireo and other five hashing-based deconvolution methods: (A) Demuxem, (B) Multiseq, (C) HashedDrops, (D) HTODemux, (E) GMM-Demux, (F) HashSolo, and (G) BFF. Vireo is fixed on the x-axis as baseline. The rows represent the hashtags while the columns represent the anonymous donor clusters genotyped by Vireo. The values within the cells represent the Pearson correlation score between singlets assigned to a specific hashtag and those assigned to a donor cluster. H) Heatmap summarising the donor matching result shows that DemuxEM and Multiseq are concordant with all genotype-based deconvolution methods except scSplit, where all the donors are matched with a good matching score. The consistency between Freemuxlet and hashedDrops can also be observed.

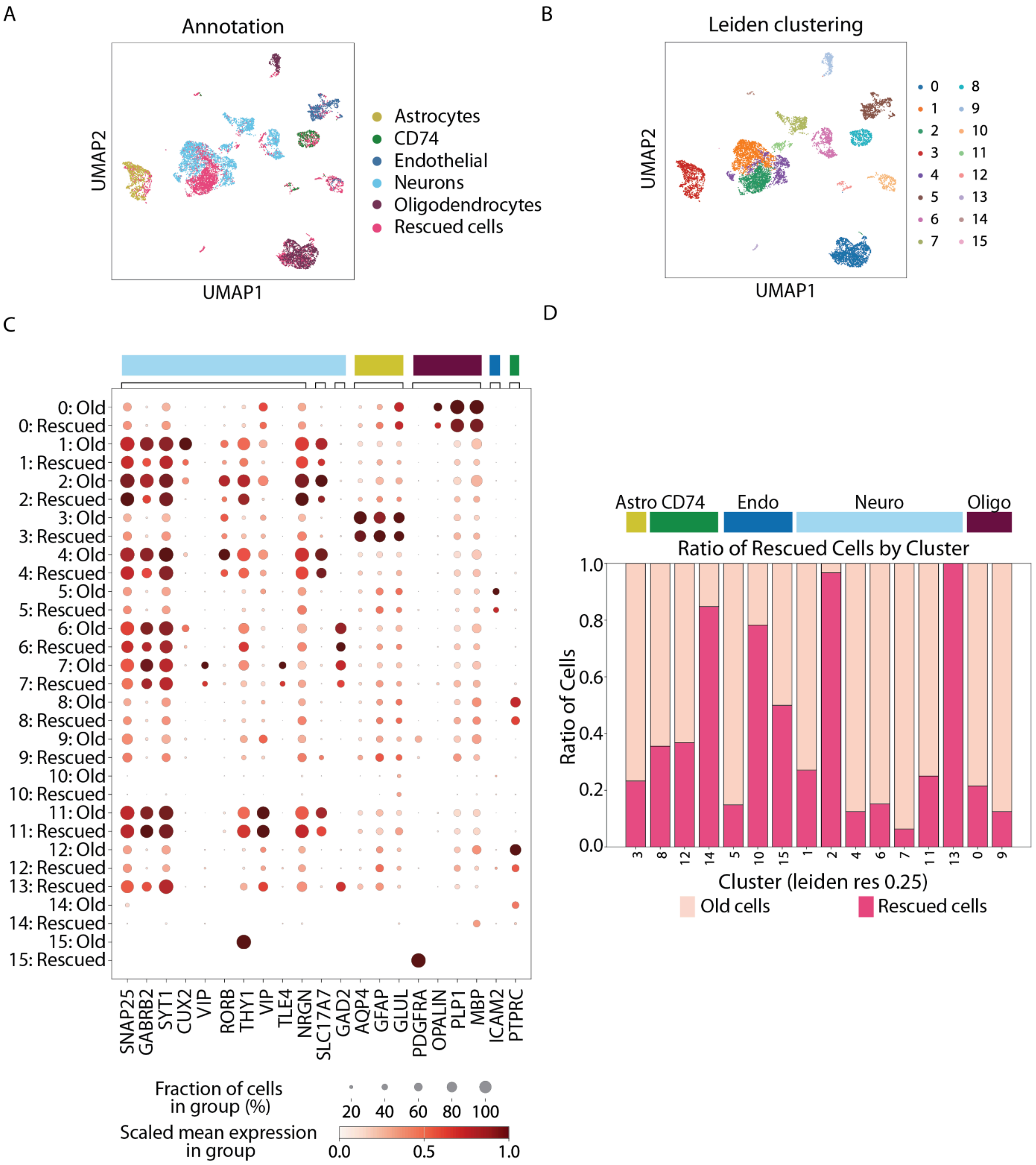

Fig. S11. Recovered cells in the second batch (gx38) of the mpxMS-dataset recapitulate original results. (A) UMAP of the single cell gene expression data with old and rescued cells. (B) Leiden clustering of the dataset with old and rescued cells. (C) Dotplot of a selection of marker genes shows concordant expression of markers in old and rescued cells. (D) Barplot showing the cluster-composition in old and rescued cells. Colours on top of barplot identify the cell annotation from (A).

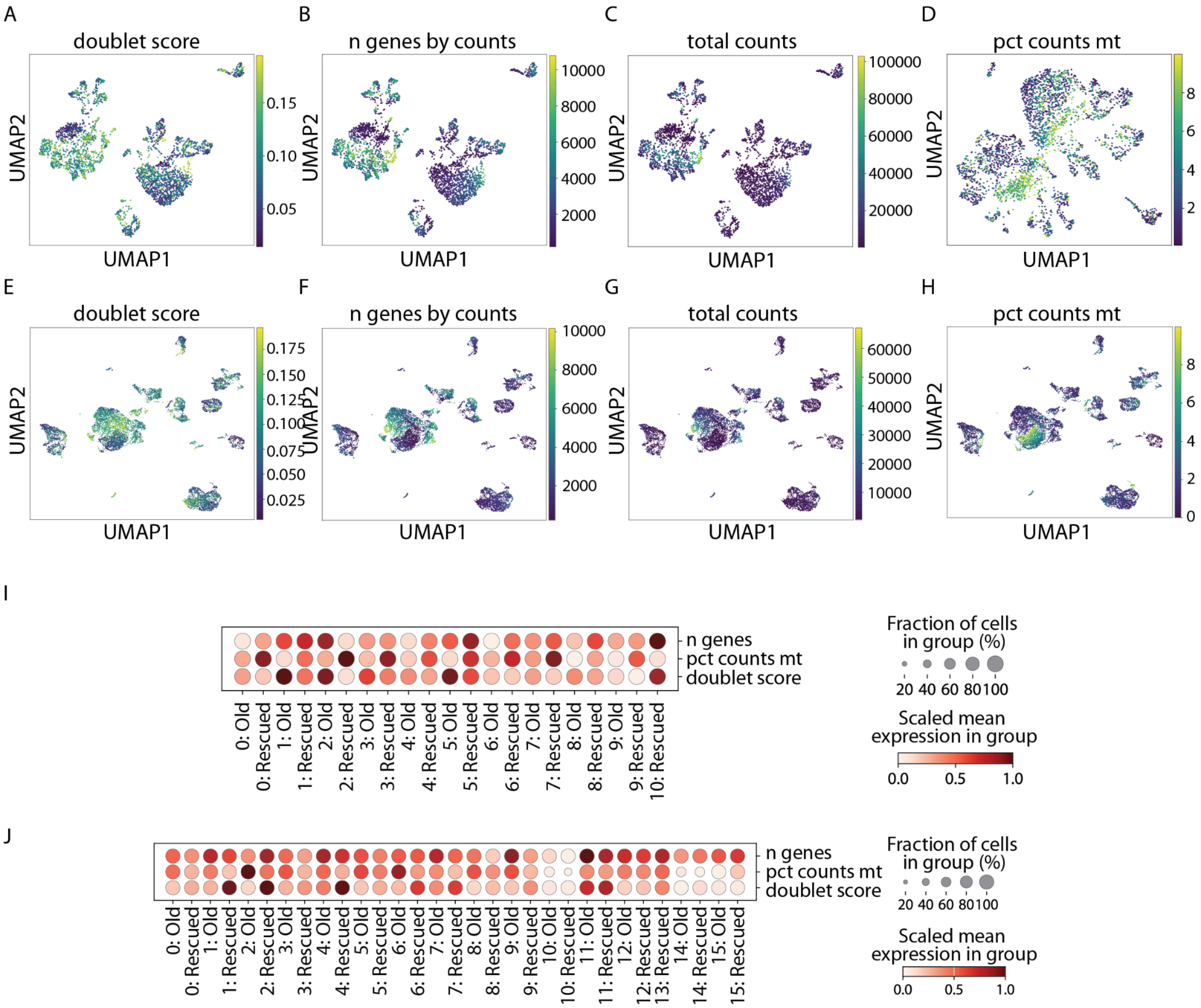

Fig. S12. Comparison of cell quality metrics between old and rescued cells in the mpxMS-dataset. (A) - (D) UMAP of the single cell gene expression data of the first batch (gx12) with old and rescued cells coloured by the number of genes by counts, the total counts and the percentage of mitochondrial reads respectively. (E) - (H) UMAP of the single cell gene expression data of the second batch (gx38) with old and rescued cells coloured by the number of genes by counts, the total counts and the percentage of mitochondrial reads respectively. (I-J) QC metrics (number of genes, percent mitochondrial reads, doublet scores) averaged by cluster and rescue state.

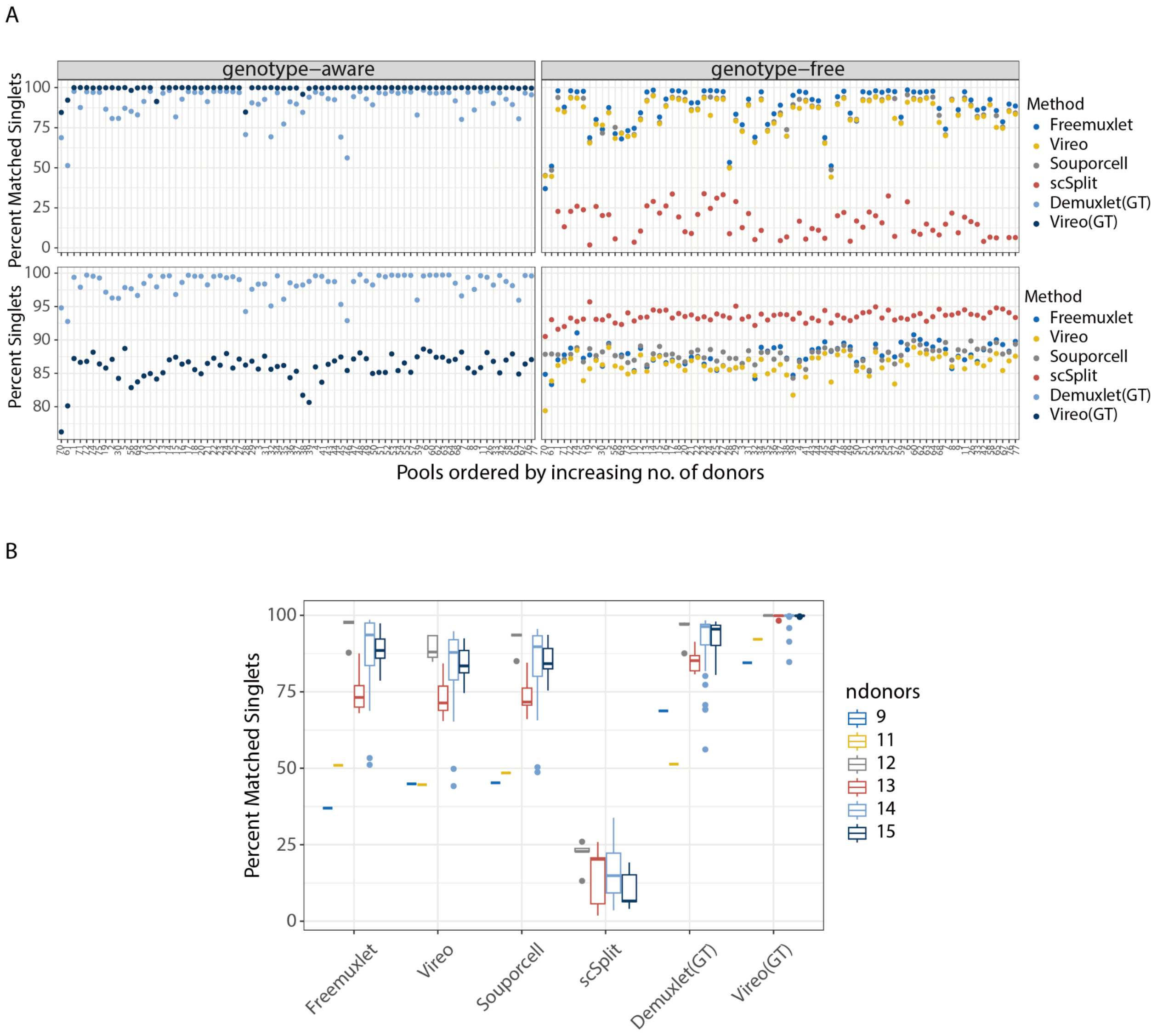

Fig. S13. Genetic deconvolution tools performance is affected by the number of donors in the pools. (A) percent matching singlets ordered by number of donors in the pool (B) Boxplots of percent matching singlets grouped by method and number of donors in the pool.

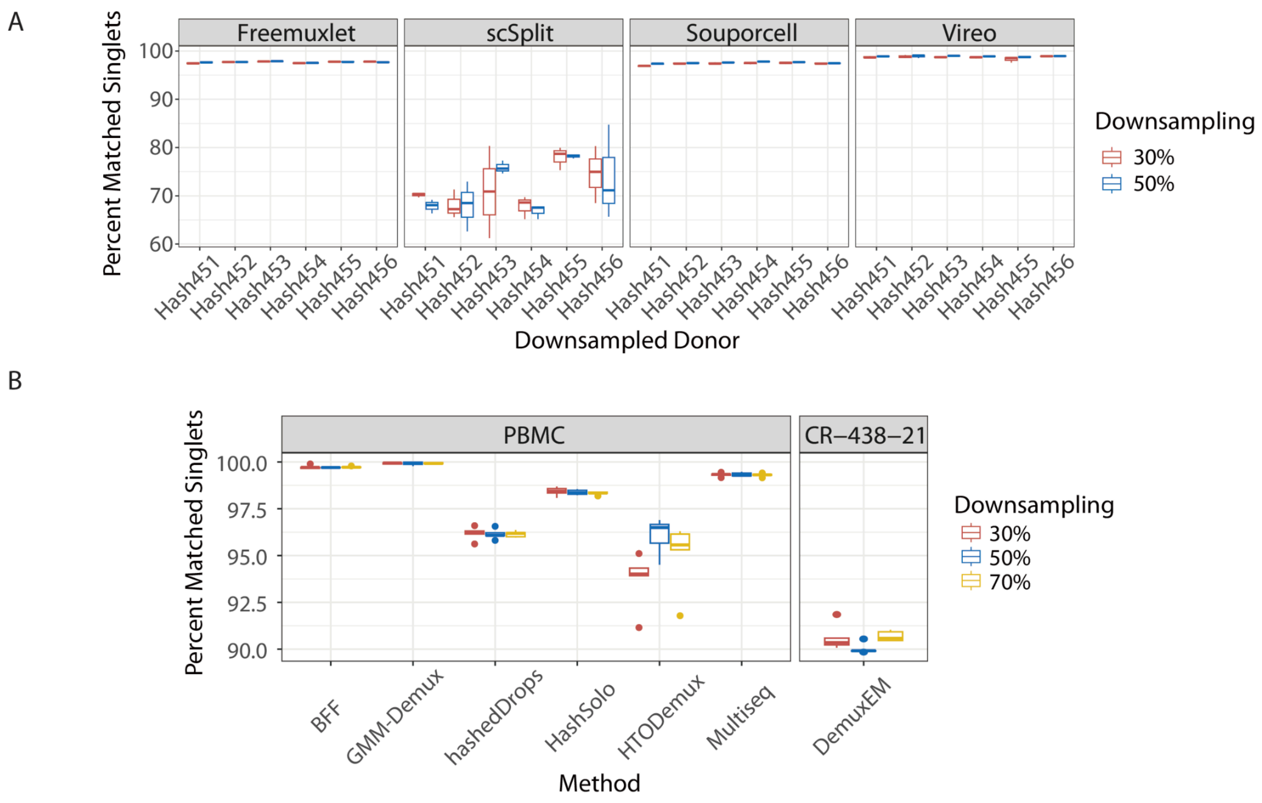

Fig. S14 Demultiplexing on downsampled datasets shows demultiplexing performance is affected by number of cells. (A) Genetic demultiplexing on the downsampled mpxMS gx12 data using three random seeds and two downsampling proportions. (B) Hashing demultiplexing on the PBMC and CR-438 dataset using five random seeds and 3 downsampling proportions.
