# Supplementary material for: hadge: a comprehensive pipeline for donor deconvolution in single-cell studies
Source: Genome Biol. 2024 Apr 26;25:109. doi: 10.1186/s13059-024-03249-z (PMC11055383; doi:10.1186/s13059-024-03249-z)
Supplement: Supplementary file 3 — Additional file 3: Supplementary tables. [file 13059_2024_3249_MOESM3_ESM.pdf]

Tool	Runtime (min): mpxMS GX12	Runtime (min): mpxMS GX38
BFF	0.67	1.82
DemuxEM	4.68	3.8
GMM-Demux	0.18	0.64
HashedDrops	2.4	6.8
HashSolo	4.28	5.35
HTODemux	0.58	1.37
Multiseq	0.40	0.65

Table S1: Runtime (in minutes) of individual hashing-based deconvolution tools applied to the mpxMS:gx12 and mpxMS:gx38 dataset.

Method	R2 ndonors	p.adj
Freemuxlet	0.16	0.002
Vireo	0.13	0.006
Souporcell	0.12	0.006
scSplit	0.05	0.05
Demuxlet(GT)	0.12	0.006
Vireo(GT)	0.2	0.0003

Table S2: R-squared values of the linear model testing for association of the percent matched singlets with number of donors for each pool.
