# Supplementary material for: hadge: a comprehensive pipeline for donor deconvolution in single-cell studies
Source: Genome Biol. 2024 Apr 26;25:109. doi: 10.1186/s13059-024-03249-z (PMC11055383; doi:10.1186/s13059-024-03249-z)
Supplement: Supplementary file 4 — Additional file 4: Review History. [file 13059_2024_3249_MOESM4_ESM.docx]

Review History

**First round of review**

**Reviewer 1**

**Were you able to assess all statistics in the manuscript, including the appropriateness of statistical tests used?**

Yes, and I have assessed the statistics in my report.

**Have you been able to install and test the software?**

Yes

**Comments to author:**

In this manuscript, Curion et. al proposed a pipeline to improve the single cell multiplexing by combining both cell hashing based and genotype based methods. Sufficient details were provided in the manuscript. The functionality of the software was clearly introduced. Benchmark and comparisons with existing methods were included. I think this manuscript can be improved by addressing the following comments:

1. In line 56 of the first page, the authors mentioned the challenge of acquisition of fresh tissue samples such as the specimens from biobank. How much would Formalin-fixed and paraffin-embedded (FFPE) tissues contribute to this challenge in cellular profiling? And what alternatives would be available to researchers? Would snRNA-seq suffer the same issue as scRNA-seq?

2. The knowledge gap is insufficiently presented. The authors mentioned no study has combined both hashing and genotype deconvolution, but it is still unclear what benefit would come out of those techniques. The description regarding potential increase in cell recovery rate and calling accurate is insufficient.

3. The choice of Pearson correlation to determine the optimal match in binary matrix is unlikely to be the optimal choice, because the distribution assumption of Pearson correlation is violated here.

4. In page 3, line 44, the authors described "Since the genotype-based deconvolution tools are run in a genotype-free model, they output their assignment in the form of anonymous donors." This description is vague and unclear to readers at this stage in hadge pipeline introduction.

5. The required input data types and formats for hadge should be clearly described in the text.

**Reviewer 2**

**Were you able to assess all statistics in the manuscript, including the appropriateness of statistical tests used?**

Yes, and I have assessed the statistics in my report.

**Have you been able to install and test the software?**

No

**Comments to author:**

Curion et al. compiled a nextflow pipeline with 12 existing methods to do hashing and genotype-based donor deconvolution for single cell sequencing data at scale. The method is timely for emerging large-scale scRNA-seq data. Below I list some clarification questions and a major concern on benchmarking with genotype ground truth for large scRNA-seq data.

My main concern is that there lacks ground truth of reference donor genotype as ground truth to evaluate the donor deconvolution results. The paper used the common consensus to evaluate methods. Why consensus is reassuring, but there is possibility that all methods may be wrong. A better benchmarking is to use large datasets with known donor genotype, such as
https://dx.doi.org/10.1101/2022.11.07.515446 (https://www.synapse.org/#!Synapse:syn31512863). Fujita et al. used two genotype-based tools included in hadge. It would be interesting to see if hadge can improve their demultiplexing and assess if hadge can handle large-scale scRNA-seq data of hundreds of donors and millions of cells.

In the hadge pipeline, there is only one doublet-detection method, Solo. Can you explain why in Fig 2 cd, multiple methods can identify doublets?

Fig 2cd does not include all methods in hadge. Why? How's hashsolo-detected doublets compare to the genotype-based deconvolution methods? They have similar singlet percentage.

**Reviewer 3**

**Were you able to assess all statistics in the manuscript, including the appropriateness of statistical tests used?**

Yes, and I have assessed the statistics in my report.

**Have you been able to install and test the software?**

Yes

**Comments to author:**

In this study, Curion et al. introduce the hadge pipeline, which offers the capability to deconvolve samples from both hashing and genetic multiplexing experiments, either independently or concurrently. The authors have demonstrated that joint deconvolution enhances the recovery of high-quality cells that might otherwise be discarded. Overall, I recognize the value of this study to the research community. However, there are several technical issues that require comprehensive resolution before considering it for publication.

Major concerns:
1. The manuscript mentions that the hadge pipeline integrates 5 genetics-based tools and 8 hashing-based tools in Figure 1. However, the actual data presented in Figure 2 shows that only 4 genetics-based tools and 5 hashing-based tools were employed. Please provide clarification on why these specific tools were chosen or add information about the performance of all the tools mentioned.
2. Figure 3B indicates that Multiseq and Demuxem demonstrate superior performance. However, when selecting the baseline for the rescue mode, Multiseq in combination with Vireo is favored over Demuxem. The manuscript should provide further details or persuasive reasons for this choice, especially considering that Demuxem exhibits a slightly higher matching score in specific numerical values in Figure 3B.
3. Figure 3 presents the design concept and principles of the rescue mode but lacks practical results. It would be valuable to provide additional information about the contributions of the rescue mode to different hashes, particularly those approaching zero, as shown in Figure 2A.
4. Figure 4 primarily focuses on the analysis of cell type distribution in the rescue mode and the presentation of marker genes. Additional information is needed regarding the comparison of cell quality metrics (e.g., n_genes_by_counts, total_counts, pct_counts_mt) between the rescue mode and the previous cell types. It is also suggested to use UMAP plots to demonstrate the accuracy of the characteristic genes of cell subtypes primarily composed of the rescue mode, confirming the existence of rescue-derived cells. Supporting these findings with relevant scholarly publications or basic analysis of publicly accessible scRNA-seq data from the same tissue would be beneficial.
5. Figure 5 highlights errors encountered by the pipeline with some raw tools, particularly Demuxmix. However, it does not delve into the underlying reasons for these errors. To comprehensively understand the pipeline's performance, it is crucial to clarify whether the errors are due to the pipeline's environment and parameters or inherent issues within the tools themselves. Additionally, tools that cannot run successfully should not be included in the pipeline, as they may inconvenience users.
6. The manuscript relies heavily on one unpublished dataset from multiple sclerosis patients. To enhance the robustness of the findings, it is recommended to incorporate more simulated data and published real data from various tissues to illustrate the accuracy of cell splitting among different tools.
7. The impact of the number of captured cells on individual deconvolution or joint deconvolution is not addressed in the article. Investigating whether the number of cells influences deconvolution outcomes would be a valuable addition.

Minor points:
1. The hadge pipeline on GitHub showcases a well-integrated toolset and good readability. However, for first-time users, it is suggested to provide example data and code in the GitHub readme, with clear instructions on how to use the pipeline.
2. The letters and numbers in Figure 3 are too small to be clearly visible.
3. In Figure 4D, the label "new" might be misinterpreted as "rescue+old." To enhance clarity, it should be standardized to match the labels in the figure legend.
4. Considering that Genome Biology is an elite journal, the figures in the PDF are not clear enough. Additionally, improvements in the color palette and font size should be carefully addressed to enhance readability.

**Authors Response**

**Point-by-point responses to the reviewers’ comments:**

Reviewer #1

In this manuscript, Curion et. al proposed a pipeline to improve the single cell multiplexing by combining both cell hashing based and genotype based methods. Sufficient details were provided in the manuscript. The functionality of the software was clearly introduced. Benchmarks and comparisons with existing methods were included. I think this manuscript can be improved by addressing the following comments:

We would like to thank the reviewer for the constructive feedback and addressed it below.

Major comments

1. In line 56 of the first page, the authors mentioned the challenge of acquisition of fresh tissue samples such as the specimens from biobank. How much would Formalin-fixed and paraffin-embedded (FFPE) tissues contribute to this challenge in cellular profiling? And what alternatives would be available to researchers? Would snRNA-seq suffer the same issue as scRNA-seq?

We recognize the importance of this aspect for readers interested in the practical applications of single-cell technologies. Therefore, we included key references that provide detailed insights into the challenges posed by fixation techniques and discuss the comparative effectiveness of single-nucleus RNA sequencing (snRNA-seq) and single-cell RNA sequencing (scRNA-seq) in this context, citing papers that have addressed this issue.

We revised the following section:

Despite considerable strides made in cellular profiling methods, the application of scRNA-seq to biomedical studies and clinical applications, which often require complex multi-sample and

multi-condition experiments, has been limited by sample throughput, cost, and susceptibility to technical variability[^2^](https://paperpile.com/c/dZD59r/76e4W). When samples can’t be acquired fresh or immediately processed after the acquisition, as may be the case for biobanks specimens, fixation techniques that allow to preserve the biological material and optimize single cell and nuclei profiling via multiplexing are a viable option[^3–6^](https://paperpile.com/c/dZD59r/fssq+spQrW+0XBy+MeuJ). In recent years, methods have emerged that allow the pooling of single cells and nuclei from individual samples[^7^](https://paperpile.com/c/dZD59r/TrhE), often relying on multiplexing techniques[^8^](https://paperpile.com/c/dZD59r/tCfA4).

The newly added references are:

-	Brown, D. V. et al. A risk-reward examination of sample multiplexing reagents for single cell RNA-Seq. Genomics 116, 110793 (2024).

-	Xu, Z. et al. High-throughput single nucleus total RNA sequencing of formalin-fixed paraffin-embedded tissues by snRandom-seq. Nat. Commun. 14, 2734 (2023).

2. The knowledge gap is insufficiently presented. The authors mentioned no study has combined both hashing and genotype deconvolution, but it is still unclear what benefit would come out of those techniques. The description regarding potential increase in cell recovery rate and calling accurate is insufficient.

We thank the reviewer for their comment, and acknowledge the need to better articulate the unique advantages of our proposed solution.

Hadge offers a user-friendly, zero-config solution for analyzing multiplexed single cell data at scale. It is a unified Nextflow pipeline that integrates 12 demultiplexing methods for hashing and genetic multiplexing protocols, offering benchmarking across methods, minimal setup, seamless scverse integration for faster downstream analysis, efficient computational resource use, and a novel joint demultiplexing strategy to enhance cell assignment quality and cost-efficiency in multi-sample experiments.

We clarified the benefits of the hashing and genetic deconvolution and the unique advantages offered by our pipeline in the introduction:

To date, there are two major protocols to generate a mixture of cells from multi-sample studies: “cell hashing” and “genotype-based multiplexing”. Cell hashing is a sample processing technique that tags the membrane or nuclei of cells in individual cell-suspension samples with unique oligonucleotide barcodes. [...] After staining or tagging, cells undergo a washing or quenching process, allowing for the safe combination of different samples into a single mixture in one tube. From this mixture, two separate sequencing libraries are created: one for single cell RNA (scRNA) and one for hashing oligos (HTO). These libraries are independently sequenced to yield two distinct single cell count matrices, corresponding respectively to scRNA and HTO data. To deconvolve the cell’s source sample, the HTO counts are processed to discover cell-barcodes positive for at least one hashtag, using cell-hashing deconvolution methods[^11,12,15,16^](https://paperpile.com/c/dZD59r/Ey7A+1TvY+jpeP+ps0tw). Cell barcodes are classified into “Singlets”, if they are positive for one tag, “Doublets”, if positive for two or more, and “Negatives”, when only a low background-noise signal is detectable (**Fig. 1A**). Cell-tagging approaches suffer from constraints such as low starting cell numbers, as these methods require washing steps that may result in cell-number loss. [...]

Genotype-based multiplexing allows the mixing of samples with unique genetic composition, where natural genetic variants serve as inherent cell-barcodes[^17^](https://paperpile.com/c/dZD59r/N0H8). Users can harness these genetic barcodes to determine the identity of each cell in the mixture. Provided a genotype reference, the scRNA reads are scanned for single nucleotide polymorphisms (SNPs) in the reference, and a table of SNP assignment to cells is produced to computationally infer the donors (**Fig. 1A**). Cell-barcodes with a genetic composition matching one donor are called “Singlets”, cell-barcodes with a mixture of at least two donors genotypes are deemed “Doublets”, and cells where the read coverage is insufficient to identify their genetic composition are “Negatives”. One limitation of this approach is the need to rely on additional data to correctly assign the cell mixtures. Users can genotype the individual samples through SNP arrays or bulk RNA-seq followed by variant calling, and then aggregate the expression values at these genomic positions for deconvolution. The same process can be conducted without genotype of origin or “genotype-free”, by piling up the mixture scRNA onto an unrelated genomic reference of genotypes such as provided by the “1000 Genome Project”. However, this approach can only deconvolve the cell mixture in the form of anonymous donors and additional processing is needed to match them to the sample of origin.

The limitations of each of these protocols can be mitigated when combining demultiplexing approaches. Experiments, where the hashing libraries are of low quality, can be rescued and successfully demultiplexed using the natural genetic variation of their RNA libraries. The combination of hashing and genetic deconvolution methods represents a viable option for combinatorial experimental design and can result in increased cell recovery rate and calling accuracy[^18^](https://paperpile.com/c/dZD59r/BCKwa). Moreover, joint demultiplexing can be a cost-effective deconvolution strategy as it further avoids having to produce sample-specific genotyping data in the form of SNP arrays or bulk sequencing methods for variant calling[^18^](https://paperpile.com/c/dZD59r/BCKwa). To date, at least nine hashing and five genotype-based deconvolution methods have been developed, each with unique strengths and weaknesses[^12,15,17,19–22^](https://paperpile.com/c/dZD59r/1TvY+0KO9+N0H8+MwXi+KYNV+jpeP+4axU). However, investigations on joint demultiplexing strategies have been limited to the combination of two specific tools instead of computationally testing the best combination of demultiplexing methods, therefore neglecting the utility of other widely used tools[^18^](https://paperpile.com/c/dZD59r/BCKwa). Although single workflows for hashing-based deconvolution and genotype-based deconvolution exist[^23^](https://paperpile.com/c/dZD59r/RzYrb)^,^[^24^](https://paperpile.com/c/dZD59r/94f5x), no study has combined all the tools for both approaches in a single comprehensive and efficient pipeline, such that both hashing and genotype deconvolution pipelines can be run in parallel on multiple samples, providing a score to discover the best methods across the board, with the final goal of maximising the number of rescued cells and increasing the confidence of deconvolution.

Therefore, there is a critical need for a unified pipeline that integrates the strengths of multiple donor deconvolution tools.

**We have also amended the discussion:**

Furthermore, applying the joint demultiplexing approach can reduce the cost of multi-sample, multi-condition experiments, when the same donors are challenged with multiple perturbations. In such cases, staining only one condition provides enough data to generate donor-specific genotypes, removing the need for additional genotyping and reducing the costs of the staining procedure.

3. The choice of Pearson correlation to determine the optimal match in binary matrix is unlikely to be the optimal choice, because the distribution assumption of Pearson correlation is violated here.

We thank the reviewer for their comment. To clarify the process for the optimal matching score, we have carried out a comparison of Pearson correlation, Spearman’s rank correlation and the Phi coefficient of a Chi-square test to determine the optimal match. We have included a notebook to support our strategy and have amended the manuscript to include more information. Briefly, to align the anonymous donors identified by two different methods, we evaluate the association between their assignment matrices, where the binary-code values indicate whether a cell is assigned to a donor (1) or to any other donor (0). While Pearson correlation is suitable for continuous variables, the chi-square test is applied to compare columns between the two matrices using a contingency table. As the variables are true dichotomies, the Pearson correlation coefficient, Spearman's Rank correlation coefficient and the phi coefficient of a Chi-square test are identical. For clarity, we rephrase the term "Pearson correlation" with "phi coefficient," yet the outcome remains unchanged. To prove our point we refer to the following notebook that summarises the experiment:

<https://github.com/theislab/hadge-reproducibility/blob/main/notebooks/coefficent_analysis.ipynb> We also adapted the following text sections:

We first define the hashing method that matches with the genetic demultiplexing method by calculating the Phi score (**Methods**). For each pair of hashing and genetic deconvolution outputs, we compute the pairwise Pearson correlation on the binarized cell classification vectors, thereby matching donors where a high correlation is observed. We then compute the matching score by summing the non-negative correlations and dividing by the number of expected donors, obtaining the degree of consistency in donor identification between any two methods.

**Methods:**

Next, the file is passed to the “donor matching” process which computes a score (Phi score) to associate an identity to the anonymous donors using the droplets where the concordance between one genetic donor and one hashtag is maximized.

4. In page 3, line 44, the authors described "Since the genotype-based deconvolution tools are run in a genotype-free model, they output their assignment in the form of anonymous donors." This description is vague and unclear to readers at this stage in hadge pipeline introduction.

We thank the reviewer for their suggestion on clarifying the outputs of the genetic demultiplexing tools that run in reference genotype-free mode. We have clarified this mode in the text and adapted the following section:

Genotype-based multiplexing allows the mixing of samples with unique genetic composition, where natural genetic variants serve as inherent cell-barcodes[^17^](https://paperpile.com/c/dZD59r/N0H8). Users can harness these genetic barcodes to determine the identity of each cell in the mixture. Provided a genotype reference, the scRNA reads

are scanned for single nucleotide polymorphisms (SNPs) in the reference, and a table of SNP assignment to cells is produced to computationally infer the donors (**Fig. 1A**). Cell-barcodes with a genetic composition matching one donor are called “Singlets”, cell-barcodes with a mixture of at least two donors genotypes are deemed “Doublets”, and cells where the read coverage is insufficient to identify their genetic composition are “Negatives”. One limitation of this approach is the need to rely on additional data to correctly assign the cell mixtures. Users can genotype the individual samples through SNP arrays or bulk RNA-seq followed by variant calling, and then aggregate the expression values at these genomic positions for deconvolution. The same process can be conducted without genotype of origin or “genotype-free”, by piling up the mixture scRNA onto an unrelated genomic reference of genotypes such as provided by the “1000 Genome Project”. However, this approach can only deconvolve the cell mixture in the form of anonymous donors and additional processing is needed to match them to the sample of origin.

[...]

Lacking prior individual genetic profiles that associate SNPs to explicit donors, genotype-based deconvolution tools assign cells to anonymous donors. Hadge de-anonymizes the donors by calculating the best match between a hashing and a genetic demultiplexing method.

5. The required input data types and formats for hadge should be clearly described in the text.

We clarified this further in the text:

The hadge pipeline features three distinct modes: *genetic, hashing* and *rescue* mode. The hashing and genetic mode are two independent workflows, and the rescue mode allows joint demultiplexing by combining the outputs of the two workflows. Different inputs are required for the two workflows, specifically:

For the hashing workflow, raw and filtered HTO and RNA counts are the minimum required input. Each of these outputs is normally generated by the cellranger pipeline, which outputs the required HTO and RNA counts in the unfiltered (raw) and filtered feature-barcode matrices in two file formats: the Market Exchange Format (MEX), and Hierarchical Data Format (HDF5). Hadge accepts the files in the MEX format.

For the genetic workflow, the minimal requirements are: the indexed sequence alignment file in BAM format along with its index (.BAI format), the barcodes of the cell-containing droplets in a TSV file, the number of expected donors in a mixture and the reference genotypes and the variants present in the pooled sample, both in VCF format. The VCF of the reference genotype can be an unrelated genomic reference to run methods in “genotype-free” mode. Optionally, if the pooled sample’s VCF is not available, we include two processes for variant calling (cellsnp and freebayes). Users can provide the mixed FASTA file to be used as input to generate the VCF file with freebayes, which is the default preprocessing for the scSplit method. All of the inputs, except for the reference VCF files, are commonly generated by the cellranger pipeline.

Moreover, we updated our documentation for hadge and refer to <https://hadge.readthedocs.io/en/latest/genetic.html> and [https://hadge.readthedocs.io/en/latest/hashing.htm](https://hadge.readthedocs.io/en/latest/hashing.html)l for explicit descriptions of the input data

together with example run commands and additional configuration and output documentation.

Reviewer #2

Curion et al. compiled a nextflow pipeline with 12 existing methods to do hashing and genotype-based donor deconvolution for single cell sequencing data at scale. The method is timely for emerging large-scale scRNA-seq data. Below I list some clarification questions and a major concern on benchmarking with genotype ground truth for large scRNA-seq data.

We would like to thank the reviewer for the constructive feedback and addressed it below.

Major comments

1. My main concern is that there lacks ground truth of reference donor genotype as ground truth to evaluate the donor deconvolution results. The paper used the common consensus to evaluate methods. Why consensus is reassuring, but there is possibility that all methods may be wrong. A better benchmarking is to use large datasets with known donor genotypes, such as <https://www.biorxiv.org/content/10.1101/2022.11.07.515446v1>. Fujita et al. used two genotype-based tools included in hadge. It would be interesting to see if hadge can improve their demultiplexing and assess if hadge can handle large-scale scRNA-seq data of hundreds of donors and millions of cells.

We thank the reviewer for their positive feedback on our pipeline and their suggestion to benchmark our pipeline on a large scale scRNAseq dataset with genotype ground truth to evaluate the donor deconvolution results. We thank the reviewer for suggesting the Fujita et al. dataset, which is deposited as raw FASTQ files in a controlled-access database. Unfortunately, we haven’t been able to secure access to this dataset in time for us to carry out the additional processing required to extract donor assignments from the raw FASTQ files, even after timely providing the necessary forms for the screening of our application. However, to perform the suggested benchmark and to avoid delays in our resubmission, we decided to use the onek1k dataset instead, a publicly available population-scale single-cell dataset which comprises >1 Mln cells across 1015 donors, multiplexed in more than 70 pools. Analogously to the Fujita et al. dataset, the onek1k has reference donor-genotypes ground truth, making both datasets very comparable and suited for the requested evaluation. The onek1k dataset further offers additional complexity with 1015 donors, multiplexed in 75 pools and a pool composition of 9-15 donors as opposed to the Fujita et al. dataset, which has 424 donors, multiplexed in 50 pools, with pools mixtures of 8 donors.

We have added the results of this benchmark in Figure 5, Supp. Fig 13-14 and the text, and include it below:

Benchmarking hadge’s runtime and robustness

To demonstrate the robustness and superior runtime of our proposed pipeline, we benchmarked its performance against two existing pipelines, demuxafy and cellHashR (**Table 1).** We submitted each pipeline on a Linux server with 32 CPU cores and 160GB of RAM memory. In all benchmarks, hadge showed superior performance with respect to the optimization of computational resources and runtime (**Fig. 5A**). Both hadge-genetic and *demuxafy* successfully executed all methods for the two mpxMS samples and an additional dataset. However, in the hashing deconvolution of the mpxMS data, some methods (*bff_cluster, bff_raw*) ran but failed to deconvolve the cells in both hadge-hashing and *cellhashR*. Additionally, one method (*demuxmix*) failed to initialise in both pipelines and as a standalone method. Hence, we excluded *demuxmix* from hadge. Notably, despite successfully running *gmm_demux* within *hadge-hashing* or when called outside the pipeline, we were not able to run cellhashR’s *gmm-demux* module.

Next, we leveraged hadge’s fast multi-sample, multi-process handling to investigate how the input number of cells affects the performance of genetic and hashing demultiplexing. We ran hadge with default parameters on the onek1k dataset, which comprises 75 pools of cell mixtures from 9-15 donors each[^9^](https://paperpile.com/c/dZD59r/Ym16u) and has ground truth donor-genotypes available. All tools detected a proportion of singlets per pool between 75 and 98% (**Fig. 5B**). However, when looking at matched donors within the singlets, the performance of the tools varied substantially, with scSplit having the lowest concordance with the original donor identities, while Demuxlet had the best performance in terms of recovered singlets and Vireo recovering the most matched singlets (**Fig. 5C**). We investigated if these two metrics were associated with the number of cells in each pool. Only Freemuxlet’s singlets percentage had a significant association with the number of cells per pool (R^2^ = 0.35, p.adj < 0.0001) (**Fig. 5D**), but all methods were significantly affected by the number of donors in the pools (**Supp.Fig. 13A, Supp. Table 3**), with the highest concordant calls reached on pools with 14 multiplexed donors (**Supp. Fig. 13B**). Across all the pools, Vireo, Demuxlet, Freemuxlet and Souporcell were mostly consistent, confirming these tools' superior performance, and as observed on the MS dataset (**Fig. 2,3**). Additionally, we ran downsampling on two hashing and one genetic multiplexing experiments and used hadge to obtain the percentages of correctly assigned singlets. The hashing methods showed an overall similar trend with the percentage of matched singlets decreasing with the number of cells, except for HTODemux and Demuxem **(Supp. Fig 14)**. All the hashing demultiplexing tools were able to correctly assign at least 90% of the cells after downsampling. The performance of the genetic demultiplexing tools was in line with what we observed on the onek1k dataset, and consistent for each tool across the different subsamples, with Vireo having the best performance across the board (~99% recovered matching singlets) (**Supp. Fig14).** Hadge allowed us to efficiently benchmark the demultiplexing performances of all the methods across the two workflows. Collectively, these results indicate that the number of donors and cells in the cell mixtures can significantly affect the number of recovered cells and the quality of the deconvolution for both families of demultiplexing methods.

*Figure 5. Benchmarking performance. (A) Hadge genetic and hashing demultiplexing pipelines were
benchmarked against demuxafy and cellhashR. The benchmark was performed on three samples for each
pairwise comparison, for a total offour samples. (mpxMS:gx12, mpxMS:gx38, demuxafy dataset, CR-438-21
dataset). (B) Results of hadge genetic on the onek1k cohort, each boxplot represents the distribution of
percentage singlets identified across 75 pools by each genetic deconvolution tool. (C) Percentage of correctly
matched singlets for each tool, each boxplot represents the distribution across 75 pools. (D) Dotplot showing
the effect of number of cells per pool on the percentage recovered singlet and matched singlets. The regression
line represents the fit of a linear model on the percentage of singlets identified by Freemuxlet (R2 0.35, p.adj
<0.0001)*

***Supplementary Fig. 13: Genetic deconvolution tools performance is affected by the number of donors in the pools. (A) percent matching singlets ordered by number of donors in the pool (B) Boxplots of percent matching singlets grouped by method and number of donors in the pool.***

**Minor comments**

**1. In the hadge pipeline, there is only one doublet-detection method, Solo. Can you explain why in Fig 2 cd, multiple methods can identify doublets?**

**We thank the reviewer for this comment. We have added further clarification on the process of demultiplexing and the identification of cells as doublets. Specifically, for cell hashing:**

**To deconvolve the cell’s source sample, the HTO counts are processed to discover cell-barcodes positive for at least one hashtag, using cell-hashing deconvolution methods**[**^11,12,15,16^**](https://paperpile.com/c/dZD59r/Ey7A+1TvY+jpeP+ps0tw)**. Cell barcodes are classified into “Singlets”, if they are positive for one tag, “Doublets”, if positive for two or more, and “Negatives”, when only a low background-noise signal is detectable.**

**and for genetic demultiplexing:**

**Cell-barcodes with a genetic composition matching one donor are called “Singlets”, cell-barcodes with a mixture of at least two donors genotypes are deemed “Doublets”, and cells where the read coverage is insufficient to identify their genetic composition are “Negatives”.**

**2. Fig 2cd does not include all methods in hadge. Why? How's hashsolo-detected doublets compare to the genotype-based deconvolution methods? They have similar singlet percentage.**

**We thank the reviewer for this comment, we have modified the figures to include all the methods supported in hadge. We report upset plots and heatmaps for the singlets and doublets identified by the hashing demultiplexing tools, including hashsolo, in Supplementary Figures 2,4-6,9.**

**In the libraries analysed, hashsolo identified 200 and 12000 doublets (mpxMS:gx38, Supplementary Figure 6E). Of the cells that were deemed singlets, hashsolo assigned them to only one (Supplementary Figure 4D, mpxMS:gx12) or two donors (Supplementary Figure 5D, mpxMS:gx38). Additionally, we include here a plot reporting the hashsolo-detected doublets compared to genotype-based deconvolution methods. The full classification of the cells is available in Supplementary Table 1.**

**Reviewer #3**

**In this study, Curion et al. introduce the hadge pipeline, which offers the capability to deconvolve samples from both hashing and genetic multiplexing experiments, either independently or concurrently. The authors have demonstrated that joint deconvolution enhances the recovery of high-quality cells that might otherwise be discarded. Overall, I recognize the value of this study to the research community. However, there are several technical issues that require comprehensive resolution before considering it for publication.**

We would like to thank the reviewer for the constructive feedback and we address the comments below.

Major comments

1. The manuscript mentions that the hadge pipeline integrates 5 genetics-based tools and 8 hashing-based tools in Figure 1. However, the actual data presented in Figure 2 shows that only 4 genetics-based tools and 5 hashing-based tools were employed. Please provide clarification on why these specific tools were chosen or add information about the performance of all the tools mentioned.

We thank the reviewer for pointing out that the figures were misleading to the number of tools actually implemented in Hadge. We have amended Figure 2 and the corresponding text.

The hadge pipeline consists of 12 deconvolution tools, including five genetics-based tools (Demuxlet[^17^](https://paperpile.com/c/dZD59r/N0H8), Freemuxlet[^26^](https://paperpile.com/c/dZD59r/nPTGR), Vireo[^22^](https://paperpile.com/c/dZD59r/4axU), scSplit [^20^](https://paperpile.com/c/dZD59r/MwXi), and Souporcell[^21^](https://paperpile.com/c/dZD59r/KYNV)), seven hashing-based tools (HTODemux[^27^](https://paperpile.com/c/dZD59r/eqBLQ), Multiseq[^12^](https://paperpile.com/c/dZD59r/1TvY) , HashedDrops[^28^](https://paperpile.com/c/dZD59r/NVSC), Demuxem[^15^](https://paperpile.com/c/dZD59r/jpeP), gmm-demux[^29^](https://paperpile.com/c/dZD59r/cGjmQ), BFF [^24^](https://paperpile.com/c/dZD59r/94f5x) and Hashsolo[^30^](https://paperpile.com/c/dZD59r/Iwvg)), and one doublet-detection method (Solo[^30^](https://paperpile.com/c/dZD59r/Iwvg)). All of these tools have been benchmarked in independent publications, and are widely used by the scientific community [^14,23,31,32^](https://paperpile.com/c/dZD59r/uswn+FXVDS+JbXE+RzYrb) .

We chose these tools because of:

1. Their popularity as evident by the number of citations and Github stars.
2. Their performance in existing benchmarks such as Howitt et al. 2023 and Cardiello et al. 2023.
3. Our desire to benchmark them further, especially in our joint deconvolution setting.

*Figure 2. Comparison of the performance of donor deconvolution methods. (A) The violin plot of raw HTO
counts shows high count levels of Hashtag 453 in cells with noisy or undetectable expression of the other HTOs.
(B) UMAP visualisation of normalised HTO counts coloured by HTODemux assignment shows poor separation of
the cells based on hashtags, with most droplets assigned to Hashtag 453. (C) Hashing-based deconvolution
methods show inconsistent assignment of cells, reported as the different proportions of cells identified as one of
either singlet, negative or doublet. (D) Genetic deconvolution tools show a more consistent assignment of the
cell mixture to singlets, doublets and negatives.*

2. Figure 3B indicates that Multiseq and Demuxem demonstrate superior performance. However, when selecting the baseline for the rescue mode, Multiseq in combination with Vireo is favored over Demuxem. The manuscript should provide further details or persuasive reasons for this choice, especially considering that Demuxem exhibits a slightly higher matching score in specific numerical values in Figure 3B.

We thank the reviewer for this comment. Demuxem and Multiseq have nearly identical results when assigning cells to individual hashtags (Figure 3 and Supp. Figs 1-2,4, Supp. Table 1-2). Therefore, using either output as a key to de-anonymize the genotype-free deconvoluted donors leads to the same results. Since the hashing deconvolution results are used only to propagate the labels to genetically-assigned singlets, we favoured a simpler and faster method to demonstrate the joint deconvolution step. Users have the option to

choose a different method based on their preference, using our matching score as a reference. To explain our choice of algorithm, we have added further justification in the text:

Notably, DemuxEM and Multiseq exhibited nearly identical performance **(Supp. Fig. 1-2,4)**, both assigning nearly 1,800 singlets, **(Supp. Table 1)** with Multiseq identifying slightly more singlets and being considerably faster than DemuxEM. **(Supp. Table 2**).

| Tool | Runtime (min): mpxMS GX12 | Runtime (min): mpxMS GX38 |
| --- | --- | --- |
| BFF | 0.67 | 1.82 |
| DemuxEM | 4.68 | 3.8 |
| GMM-Demux | 0.18 | 0.64 |
| HashedDrops | 2.4 | 6.8 |
| HashSolo | 4.28 | 5.35 |
| HTODemux | 0.58 | 1.37 |
| Multiseq | 0.40 | 0.65 |

*Supplementary Table 2: Runtime (in minutes) of individual hashing-based deconvolution tools applied to the
mpxMS:gx12 and mpxMS:gx38 dataset.*

Based on the observed high matching score and the successful matching of all anonymous donors (**Supp. Fig. 4-5, 10**), two hashing demultiplexing methods performed best compared to Vireo, namely Multiseq and Demuxem, both recovering identical matches between genetic and hashing donors (**Fig. 3B, Supp. Fig 4-5 and Supp Table 1**).

3. Figure 3 presents the design concept and principles of the rescue mode but lacks practical results. It would be valuable to provide additional information about the contributions of the rescue mode to different hashes, particularly those approaching zero, as shown in Figure 2A.

We thank the reviewer for this comment. We have amended Fig 3 to include more informative plots which should clarify the number of negative cells that are rescued (Figure 3E) and the number of cells that are assigned to individual donors when applying the optional refining step (Figure 3F).

*Figure 3. Joint deconvolution recovers high-quality cells. (A) Overview of the steps to extract cell-variants from
common SNPs in the population based on the assignment of Multiseq and Vireo. (B) Heatmap summarising the
donor matching result shows that DemuxEM and Multiseq are in high concordance with all genotype-based
deconvolution methods, where all the donors are matched with a high matching score. (C) Correlation heatmap
of donor identification between Vireo and Multiseq. (D) Sankey plot summarising the percentages of cells
deconvoluted by hashing (Multiseq) and after the joint deconvolution step (Vireo). (E) Number of donor-specific
variants used as input for the refinement step (F) Sankey plot summarising the percentages of cells
deconvoluted by hashing (Multiseq) and after the refinement step.*

4. Figure 4 primarily focuses on the analysis of cell type distribution in the rescue mode and the presentation of marker genes. Additional information is needed regarding the

comparison of cell quality metrics (e.g. number of genes by counts, the total counts and the percentage of mitochondrial reads) between the rescue mode and the previous cell types. It is also suggested to use UMAP plots to demonstrate the accuracy of the characteristic genes of cell subtypes primarily composed of the rescue mode, confirming the existence of rescue-derived cells. Supporting these findings with relevant scholarly publications or basic analysis of publicly accessible scRNA-seq data from the same tissue would be beneficial.

We thank the reviewer for this comment. The goal of our manuscript was to demonstrate the utility of the hadge pipeline in deconvolving cell mixtures and rescuing good-quality single cells when applying joint deconvolution methods. We apply quality filtering as recommended by state of the art best practices in single cell data analysis (Heumos & Schaar et al. 2023). Additionally, we decided to use the same filtering criteria, with a more stringent filter on doublet scores, that were applied in the original publication for the MS datasets used in this work (Grant-Peters et al. 2022). We then annotated the cells with the detailed original marker identification list provided by Grant-Peters et al. 2022, with marker genes collected from Jäkel et al., Nature 2019 and Schirmer et al., Nature 2019.

We report additional quality control plots in Supplementary Figure 12 and include the notebooks used for the data analysis step in [https://github.com/theislab/hadge-reproducibility/blob/main/notebooks/revised_gx38_analysis_LH](https://github.com/theislab/hadge-reproducibility/blob/main/notebooks/revised_gx38_analysis_LH.ipynb) [.ipynb](https://github.com/theislab/hadge-reproducibility/blob/main/notebooks/revised_gx38_analysis_LH.ipynb) and [https://github.com/theislab/hadge-reproducibility/blob/main/notebooks/revised_gx12_analysis_LH](https://github.com/theislab/hadge-reproducibility/blob/main/notebooks/revised_gx12_analysis_LH.ipynb) [.ipynb](https://github.com/theislab/hadge-reproducibility/blob/main/notebooks/revised_gx12_analysis_LH.ipynb)

*Supplementary Fig. 12: Comparison of cell quality metrics between old and rescued cells in the mpxMS-dataset. (A) - (D) UMAP of the single cell gene expression data of the first batch (gx12) with old and rescued cells colored by the number of genes by counts, the total counts and the percentage of mitochondrial reads respectively. (E) - (H) UMAP of the single cell gene expression data of the second batch (gx38) with old and rescued cells coloured by the number of genes by counts, the total counts and the percentage of mitochondrial reads respectively. (I-J)*

*QC metrics (number of genes, percent mitochondrial reads, doublet scores) averaged by cluster and rescue*

*state.*

5. Figure 5 highlights errors encountered by the pipeline with some raw tools, particularly Demuxmix. However, it does not delve into the underlying reasons for these errors. To comprehensively understand the pipeline's performance, it is crucial to clarify whether the errors are due to the pipeline's environment and parameters or inherent issues within the tools themselves. Additionally, tools that cannot run successfully should not be included in the pipeline, as they may inconvenience users.

The errors and failures of the pipeline are explicitly not because of our implementation but because of bugs in the tools, particularly Demuxmix. We contacted the authors of Demuxmix for assistance to get their tool running, but we were unable to make it work in time for the resubmission of this manuscript. We agree with the reviewer that tools that cannot be run successfully should be removed from the pipeline and therefore we removed Demuxmix.

We added reasoning in the text:

Additionally, one method (demuxmix) failed to initialise in both pipelines and as a standalone method. Hence, we excluded demuxmix from hadge.

6. The manuscript relies heavily on one unpublished dataset from multiple sclerosis patients. To enhance the robustness of the findings, it is recommended to incorporate more simulated data and published real data from various tissues to illustrate the accuracy of cell splitting among different tools.

We thank the reviewer for suggesting to benchmark our tool on additional datasets. We have additionally ran hadge on the onek1k dataset, and on two additional hashing datasets, also applying downsampling to simulate varying number of cells. We discuss the results in Figure 5 and in the text (see next section)

7. The impact of the number of captured cells on individual deconvolution or joint deconvolution is not addressed in the article. Investigating whether the number of cells influences deconvolution outcomes would be a valuable addition.

We thank the reviewer for suggesting to evaluate the performance of demultiplexing tools when dealing with different cell numbers. To evaluate this scenario, we run hadge on the onek1k dataset, and applied downsampling to two hashing and one genetic demultiplexing samples. We summarise our findings in Figure 5and Supplementary Figures 13-14 and for clarity include them below:

[...] Next, we leveraged hadge’s fast multi-sample, multi-process handling to investigate how the input number of cells affects the performance of genetic and hashing demultiplexing. We ran hadge with default parameters on the onek1k dataset, which comprises 75 pools of cell mixtures from 9-15 donors each[^9^](https://paperpile.com/c/dZD59r/Ym16u) and has ground truth donor-genotypes available. All tools detected a proportion of singlets per pool between 75 and 98% (**Fig. 5B**). However, when looking at matched donors within the singlets, the performance of the tools varied substantially, with scSplit having the lowest concordance with the original donor identities, while Demuxlet had the best performance in terms of recovered singlets and Vireo recovering the most matched singlets (**Fig. 5C**). We investigated if these two metrics were associated with the number of cells in each pool. Only Freemuxlet’s singlets percentage had a significant association with the number of cells per pool (R^2^ = 0.35, p.adj < 0.0001) (**Fig. 5D**), but all methods were significantly affected by the number of donors in the pools (**Supp.Fig. 13A, Supp. Table 3**), with the highest concordant calls reached on pools with 14 multiplexed donors (**Supp. Fig. 13B**). Across all the pools, Vireo, Demuxlet, Freemuxlet and Souporcell were mostly consistent, confirming these tools' superior performance, and as observed on the MS dataset (**Fig. 2,3**). Additionally, we ran downsampling on two hashing and one genetic multiplexing experiments and used hadge to obtain the percentages of correctly assigned singlets. The hashing methods showed an overall similar trend with the percentage of matched singlets decreasing with the number of cells, except for HTODemux and Demuxem **(Supp. Fig 14)**. All the hashing demultiplexing tools were able to correctly assign at least 90% of the cells after downsampling. The performance of the genetic demultiplexing tools was in line with what we observed on the onek1k dataset, and

consistent for each tool across the different subsamples, with Vireo having the best performance across the board (~99% recovered matching singlets) (**Supp. Fig14).** Hadge allowed us to efficiently benchmark the demultiplexing performances of all the methods across the two workflows. Collectively, these results indicate that the number of donors and cells in the cell mixtures can significantly affect the number of recovered cells and the quality of the deconvolution for both families of demultiplexing methods.

*Figure 5. Benchmarking performance. (A) Hadge genetic and hashing demultiplexing pipelines were
benchmarked against demuxafy and cellhashR. The benchmark was performed on three samples for each
pairwise comparison, for a total offour samples. (mpxMS:gx12, mpxMS:gx38, demuxafy dataset, CR-438-21
dataset). (B) Results of hadge genetic on the onek1k cohort, each boxplot represents the distribution of
percentage singlets identified across 75 pools by each genetic deconvolution tool. (C) Percentage of correctly
matched singlets for each tool, each boxplots represents the distribution across 75 pools. (D) Dotplot showing
the effect of number of cells per pool on the percentage recovered singlet and matched singlets. The regression
line represents the fit of a linear model on the percentage of singlets identified by Freemuxlet (R2 0.35, p.adj
<0.0001)*

*Supplementary Fig. 13: Genetic deconvolution tools performance is affected by the number of donors in the
pools. (A) percent matching singlets ordered by number of donors in the pool (B) Boxplots of percent matching
singlets grouped by method and number of donors in the pool.*

***Supplementary Fig. 14 Demultiplexing on downsampled datasets shows demultiplexing performance is affected
by number of cells. (A) Genetic demultiplexing on the downsampled mpxMS gx12 data using three random
seeds and two downsampling proportions. (B) Hashing demultiplexing on the PBMC and CR-438 dataset using
five random seeds and 3 downsampling proportions.***

**Methods**

**Cell number downsampling experiments**

**We performed consecutive downsampling of two publicly available hashing datasets, a PBMC multiplexed sample with 4 hashtags and a total of 15843 cells (**[**https://www.ncbi.nlm.nih.gov/geo/query/acc.cgi?acc=GSE152981**](https://www.ncbi.nlm.nih.gov/geo/query/acc.cgi?acc=GSE152981)**) and the test data used in the cellHashR pipeline, hereby called CR-438-21, for a total of 11090 cells (**[**https://github.com/BimberLab/cellhashR/tree/master/tests/testdata/438-21-GEX**](https://github.com/BimberLab/cellhashR/tree/master/tests/testdata/438-21-GEX)**) which also provides raw rna data to enable running demuxem. The same dataset was also used for the run time benchmarks (see “Run time benchmarks”). For each sample, we applied random downsampling to 30-50-70% of the cell barcodes in the HTO matrix, using five different seeds to control for performance variations. The downsampled counts were then fed to the hadge hashing workflow and each method was run under default conditions. For the PBMC dataset, the ground truth labels were obtained running gmm-demux on the full sample as described in the original publication**[**^29^**](https://paperpile.com/c/dZD59r/cGjmQ)**. For the CR-438-21, the ground truth labels were obtained using the instructions provided on the cellhashR repository. The scripts used for downsampling are available on** [**https://github.com/theislab/hadge-reproducibility/**](https://github.com/theislab/hadge-reproducibility/)

Downsampling of the genetic data was performed on the first batch (gx12) of mpxMS-dataset, using as ground truth the joint deconvolution results between vireo and multiseq demonstrated in Figs. 3-4. We performed random downsampling to 30% and 50% of the cell barcodes associated with each donor, using three different seeds, and evaluated the percentage matching singlets recovered after removing 30 or 50% of the cells for a particular donor across the different tested seeds. To reduce the run time, bam files were pre-processed to contain only reads that overlap with known common SNPs from the 1000 Genomes Project (<https://sourceforge.net/projects/cellsnp/files/SNPlist/>). The downsampled reads were then used as input for hadge genetic workflow and each method was run under default conditions.

Minor comments

1. The hadge pipeline on GitHub showcases a well-integrated toolset and good readability. However, for first-time users, it is suggested to provide example data and code in the GitHub readme, with clear instructions on how to use the pipeline.

We strongly agree with the author that software should be well-documented with clear examples. We improved hadge in this regard by:

- Adding a quickstart section in the README that downloads example data and runs the hadge pipeline with it.
- Greatly expanding our documentation on ReadTheDocs
  (<https://hadge.readthedocs.io/en/latest/>) which now features:
- More detailed installation and run instructions
- More details on the output including an example notebook that shows how the output can be analyzed
- Detailed sections on the two main approaches (cell hashing and genetic
  demultiplexing) with detailed tool,parameter, and output descriptions
- Detailed explanation of the ‘rescue’ mode

2. The letters and numbers in Figure 3 are too small to be clearly visible.

We increased and harmonized the font size in all of our figures to make them more readable.

*Figure 3. Joint deconvolution recovers high-quality cells. (A) Overview of the steps to extract cell-variants from
common SNPs in the population based on the assignment of Multiseq and Vireo. (B) Heatmap summarizing the
donor matching result shows that DemuxEM and Multiseq are in high concordance with all genotype-based
deconvolution methods, where all the donors are matched with a high matching score. (C) Correlation heatmap
of donor identification between Vireo and Multiseq. (D) Sankey plot summarising the percentages of cells
deconvoluted by hashing (Multiseq) and after the joint deconvolution step (Vireo). (E) Number of donor-specific
variants used as input for the refinement step (F) Sankey plot summarising the percentages of cells
deconvoluted by hashing (Multiseq) and after the refinement step.*

3. In Figure 4D, the label "new" might be misinterpreted as "rescue+old." To enhance clarity, it should be standardised to match the labels in the figure legend.

We thank the reviewer for this suggestion and apologise for the misleading labels used in Figure 4. We have amended the Figure and removed the “new” label, and now use only the term “rescued” to refer to the cells that are rescued by joint deconvolution as described in the text.

*Figure 4. Recovered cells recapitulate known cell types (A) UMAP of the single cell gene expression data with
old and rescued cells. (B) Leiden clustering of the dataset with old and rescued cells. (C)* SYT1 *expression defines
rescued cells as a new cluster of neurons. (D) Dotplot of a selection of marker genes shows concordant
expression of markers in old and rescued cells. (E) Barplot showing the cluster-composition in old and rescued
cells, with two neuronal clusters enriched for rescued cells. Colors on top of barplot identify the cell annotation
from (A).*

4. Considering that Genome Biology is an elite journal, the figures in the PDF are not clear
enough. Additionally, improvements in the color palette and font size should be carefully
addressed to enhance readability.

We took the following measures general to improve our figures:

1. We increased the font size and harmonized the font across all figures.

**2. We are now using a consistent colour palette for hashing (violet) and genetic (green) deconvolution across all figures and we further harmonized colours for all other plots wherever possible to reduce the number of colours.**

**Second round of review**

**Reviewer 1**

The authors have sufficiently addressed my questions.

**Reviewer 2**

My comments have been addressed. I have two minor questions/suggestions:

1. The title "hadge: a comprehensive pipeline for donor deconvolution in single cell": it may be more precise to use "single cell studies" or "single cell sequencing", other than "single cell" alone in a vague way.

2. Fig 5 in the manuscript and response does not match exactly. I do not know why.

**Reviewer 3**

All my concerns have been nicely addressed.

**Authors Response**

**Point-by-point responses to the reviewers’ comments:**

**Reviewer #2**

My comments have been addressed. I have two minor questions/suggestions:
1. The title "hadge: a comprehensive pipeline for donor deconvolution in single cell": it may be more precise to use "single cell studies" or "single cell sequencing", other than "single cell" alone in a vague way.

>Thank you for your suggestion. We adapted the title to “hadge: a comprehensive pipeline for donor deconvolution in single cell studies”

2. Fig 5 in the manuscript and response does not match exactly. I do not know why.

>The figure used in the response is the correct version. We now use the correct version in the manuscript.
